# Subclonal evolution in disease progression from MGUS/SMM to multiple myeloma is characterised by clonal stability

**DOI:** 10.1038/s41375-018-0206-x

**Published:** 2018-07-25

**Authors:** Ankit K. Dutta, J. Lynn Fink, John P. Grady, Gareth J. Morgan, Charles G. Mullighan, Luen B. To, Duncan R. Hewett, Andrew C. W. Zannettino

**Affiliations:** 1 Myeloma Research Laboratory, Adelaide Medical School, Faculty of Health and Medical Sciences, The University of Adelaide, Adelaide, SA 5005 Australia; 2 Cancer Theme, South Australian Health and Medical Research Institute (SAHMRI), Adelaide, SA 5000 Australia; 3 Genomic Medicine Division, The University of Queensland, Diamantina Institute (UQDI), Brisbane, QLD 4102 Australia; 4 The Myeloma Institute, University of Arkansas for Medical Sciences, Little Rock, AR 72205 USA; 5 Department of Pathology and the Hematological Malignancies Program, St Jude Children’s Research Hospital, Memphis, TN 38105 USA; 6 SA Pathology, Adelaide, SA 5000 Australia; 7 Haematology and Bone Marrow Transplant Unit, Royal Adelaide Hospital, Adelaide, SA 5000 Australia

**Keywords:** Cancer genomics, Genetics research, Myeloma, Cancer genomics

## Abstract

Multiple myeloma (MM) is a largely incurable haematological malignancy defined by the clonal proliferation of malignant plasma cells (PCs) within the bone marrow. Clonal heterogeneity has recently been established as a feature in MM, however, the subclonal evolution associated with disease progression has not been described. Here, we performed whole-exome sequencing of serial samples from 10 patients, providing new insights into the progression from monoclonal gammopathy of undetermined significance (MGUS) and smouldering MM (SMM), to symptomatic MM. We confirm that intraclonal genetic heterogeneity is a common feature at diagnosis and that the driving events involved in disease progression are more subtle than previously reported. We reveal that MM evolution is mainly characterised by the phenomenon of clonal stability, where the transformed subclonal PC populations identified at MM are already present in the asymptomatic MGUS/SMM stages. Our findings highlight the possibility that PC extrinsic factors may play a role in subclonal evolution and MGUS/SMM to MM progression.

## Introduction

Multiple myeloma (MM) is a haematological malignancy characterised by the uncontrolled proliferation of neoplastic plasma cells (PCs) within the bone marrow (BM). MM accounts for ~10% of all haematological malignancies [1], with a median survival rate of 5.2 years [2]. Although ten new therapeutic agents for MM have been approved in the last 20 years, and patient outcomes have improved significantly, individual responses to therapy and overall survival are varied [3]. MM remains largely incurable, with relapse being a common feature of disease.

The development of MM has been classically viewed as a multistage process involving the acquisition of multiple genetic mutations, with immunoglobulin heavy chain translocations and hyperdiploidy known to be common initiating events that deregulate normal PC behaviour leading to the development of monoclonal gammopathy of undetermined significance (MGUS) [4–7]. Further mutational load leads to an intermediate stage of smouldering multiple myeloma (SMM) [4, 8]. However, these common initiating events are insufficient to cause MM transformation, as MGUS/SMM patients commonly harbour these abnormalities and show no clinical symptoms of MM [9, 10]. Studies have shown that progression to MM is associated with additional genetic changes including aneuploidy, chromosomal translocations, single nucleotide variants, small insertions and deletions, and copy number variants affecting one or more genes, with mutations present at a frequency of 0.1 to 10 per megabase [11].

Recent studies show that MM patients display complex mutational landscapes involving intraclonal genetic heterogeneity at the bulk tumour level, where mutations are acquired in a non-linear branching pattern [12–17]. Intraclonal heterogeneity has been observed at all stages of MM, suggesting that disease progression may be mediated through inter-subclone competition and outgrowth of the fittest of these subclones. Genomic studies on large cohorts of unmatched MGUS-SMM-MM patients have led to the discovery of recurrently mutated genes, of which *KRAS*, *NRAS*, *TP53*, *BRAF*, *FAM46C* and *DIS3* are believed to be drivers of MM transformation [10, 18–21]. While clonal heterogeneity is now an established feature in MM, the subclonal evolution associated with MGUS/SMM to MM progression remains poorly understood.

A comprehensive approach to identifying the key somatic mutations and infer the subclonal evolution associated with MM transformation, involves the longitudinal study of sequential MGUS-MM or SMM-MM samples from the same patient. However, because MGUS is often an incidental finding, it is extremely rare to have diagnostic BM samples from the same patient at both the MGUS and MM stages. In addition, because there are no cell line or mouse models of MGUS or SMM, there has been limited opportunity to study the specific genetic changes and molecular mechanisms that characterise the progression from MGUS/SMM to MM.

Here, we report a longitudinal genomics investigation of MM, based on paired MGUS-MM (*n* = 5) or SMM-MM (*n* = 5) patient samples obtained from the same patient when initially diagnosed at MGUS/SMM, and subsequently when they developed MM. Using whole-exome sequencing, we have obtained a detailed description of the genomic complexity and subclonal evolution underlying progression from MGUS/SMM to MM.

## Materials and methods

### Clinical samples

Bone marrow mononuclear cell aspirates were collected from patients at MGUS/SMM, and subsequently at later diagnosis of MM (MGUS-MM (*n* = 5) and SMM-MM (*n* = 5)). The median time to progression of MGUS to MM was 3.2 years (range 1–13 years) and SMM to MM was 1.2 years (range 0.48–4.1 years). Where available, the cytogenetic status of patients is reported in Supplementary Table 1. Samples were collected from patients prior to treatment. All patients provided informed consent in accordance with the Declaration of Helsinki. Samples were cryopreserved by the South Australian Cancer Research Biobank (SACRB) at SA Pathology. The studies were approved by the Royal Adelaide Hospital Human Research Ethics Committee (HREC/13/RAH/569 No: 131133). Samples were collected over a period of 22 years, and we initially began this study with paired-samples from 18 patients. However, due to our strict criteria for sample purity and mutation calling resolution, final analysis was only performed on samples from 10 patients.

### Cell sorting

PCs and non-tumour cells were purified using multicolour flow cytometry as previously described [21]. Briefly, ~1 × 10^5^ mononuclear cells were prepared for single-stain antibody control (CD138-PE (Beckman Coulter #A54190) and CD38-PE-Cy7 (Biolegend #303515)) and compensation/FMO tubes (1: unstained; 2: hydroxystilbamidine (FluoroGold; Life Technologies) only; 3: CD38-PE-Cy7 + FluroGold; 4: CD138-PE + FluroGold; and 5: CD38-PE-Cy7 + CD138-PE). The sort sample was stained with CD138-PE and CD38-PE-Cy7 antibody at 1 μL/100 μL cells. Cells were stained with FluoroGold immediately prior to sorting. Viable PCs (CD138^+^CD38^+^ and FluoroGold negative) and non-tumour cells were sorted on the FACSAria Fusion sorter (BD Biosciences). FACS purity check was carried out on sorted cells, using 100–500 cells from each sample.

### DNA isolation, QC and sequencing

DNA was isolated from purified PC and non-tumour populations using the All Prep DNA/RNA Micro Kit (QIAGEN) as per manufacturers’ instructions. Yields and quality was assessed using the NanoDrop 8000 and Qubit 2.0 fluorometer (Thermo Fisher Scientific).

A total of 115 ng of gDNA was used as an input for fragmentation on the Covaris E220, followed by end-repair/A-tailing and ligation of SureSelect Adapter Oligos (Agilent). Pre-Capture PCR amplification of 10 cycles, or 12 cycles for low input samples, were performed. A total of 750 ng of each sample was hybridised to SureSelect XT Clinical Research Exome (Agilent) probes overnight. Captured DNA was amplified with 11 cycles of post-capture PCR incorporating index barcodes. Sequencing was performed on the Illumina HiSeq4000 (2 × 100 bp paired-end reads) and NextSeq 500 (2 × 150 bp paired-end reads). Samples were sequenced to a minimum depth of ~140 × mean coverage. Isolated non-tumour cells were also sequenced to a similar average depth (138x).

### Analysis of data

Sequencing reads were mapped to the human decoy genome (hs37d5) using Novoalign (v3.02.08), followed by post-processing according to GATK best practices [22]. Somatic single nucleotide and small indel variants were called using MuTect2 [23] and multiSNV [24]. Variants were filtered based on: 10+ reads covering the variant site; 5+ reads covering the variant in the tumour sample. Variants were annotated with SnpEff [25].

R 3.3.2 was used throughout for analyses. Somatic copy number variants were called using CNVkit [26] v0.7.11 and custom in-house methods developed to support highly aneuploid genomes to perform segmentation and calculate log2 changes.

Clonal evolution was investigated using PhyloWGS [27] and visualised using fish plot in R [28]. PhyloWGS is noted to inflate the number of subclones, thus we recognise that subclone numbers may be overestimated. All phylogenetic trees constructed were based on the assumption that there is a single founder clone.

Additional information on sequencing and somatic mutation analysis is given in the supplementary methods.

### Data deposition

All raw sequencing reads have been deposited in the EGA repository (Accession number: EGAS00001002850).

### Code availability

Custom script generated for CNV analysis is available on request from corresponding authors.

## Results

### A changing spectrum of acquired mutations, not mutational load, is associated with MM progression

Whole-exome sequencing was performed on paired MGUS/SMM to MM patients (detailed in Supplementary Table 1) to a minimum average depth of 140x (Supplementary Table 2). A total of 1614 somatic non-synonymous single nucleotide variants (NS-SNVs) were identified across the MGUS/SMM samples (range 30–220) with a median 161 per patient. Interestingly, in the MM samples, we identified a total 1508 somatic NS-SNVs (range 59–226), with a median 152 per patient. There was an average of 27 NS-SNVs that were shared between the MGUS/SMM and MM stage (range 0–53) (Supplementary Table 3). We observed a moderately higher mutation load compared to previous larger cohort studies of MM, which identified median SNV numbers of 31 (range 15–46) [4], and 52 mutations per patient (range 2–488) [18].

**Fig. 1:**
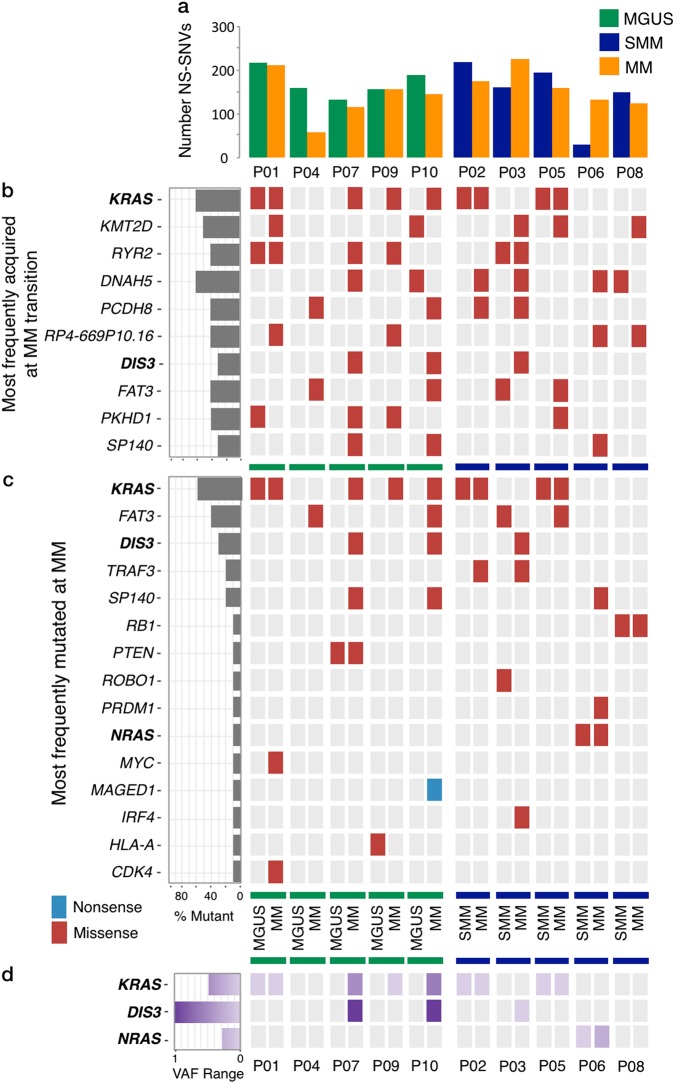
Pattern of genetic mutations in MGUS/SMM to MM progression. **a** Total NS-SNV mutational load associated with progression from MGUS/SMM to MM in individual patients. **b** Waterfall diagram indicating the 10 most frequently mutated genes associated with progression from MGUS/SMM to MM. **c** We identified mutations in 15 reported recurrently mutated MM genes in our MGUS/SMM to MM samples. However, individual patients harbour a heterogeneous genetic architecture, with a combination of mutations in known driver (*KRAS*, *NRAS* and *DIS3*) and other candidate genes. Mutations in RAS/MAPK pathway genes are most prevalent. **d** Gradient diagram across all patients indicating the variant allele frequencies (VAF) of identified known cancer drivers. RAS mutations are observed to mainly occur subclonally

Recent sequencing studies of unpaired MM samples have described an increasing median NS-SNV burden from MGUS to SMM to MM, with MGUS patients harbouring approximately half the number of NS-SNVs when compared to unmatched MM patients [4], with an average of 35 at the MM stage [10]. Here, we observed the opposite upon progression from MGUS/SMM to MM, where seven out of ten patients showed a decrease in total mutational load (Fig. 1a). While the total mutational burden is not considerably different between MGUS/SMM and MM, the presence of intraclonal heterogeneity and changes in the spectrum of mutated genes between disease stages, suggests that there is waxing and waning of subclones over time [29].

We next examined the changing mutational landscape associated with MGUS/SMM to MM progression, to identify the genetic aberrations associated with this process, including both previously reported ‘drivers’ of MM and frequently acquired mutations present at MM transition. To this end, we identified 2566 unique genes with acquired variants at MM transition across all patients. The most common genes harbouring mutations at MM include *KRAS*, *KMT2D*, *RYR2*, *DNAH5*, *PCDH8*, *RP4-669P10.16*, *DIS3*, *FAT3*, *PKHD1* and *SP140* (Fig. 1b). Moreover, we identified 15 previously reported recurrently mutated genes: *KRAS*, *FAT3, DIS3, TRAF3, SP140, RB1, PTEN, ROBO1, PRDM1, NRAS, MYC, MAGED1, IRF4, HLA-A* and *CDK4* [10, 18–20, 30] (Fig. 1c), including mutations in three known ‘drivers’ of MM: *KRAS*, *NRAS* and *DIS3* (Supplementary Table 4). In our samples, mutations in *KRAS* and *NRAS*, were mutually exclusive, consistent with previous observations that report the rare co-occurrence of mutations in these genes (in 2% of patients) [20].

The RAS/MAPK pathway was highly mutated with 40% of patients at MGUS/SMM, and 70% at MM, harbouring mutations in *KRAS* and *NRAS*. *DIS3* was mutated in 30% of patients at the MM stage only (Fig. 1d). This highlights that driver mutations can be acquired at both the asymptomatic stages and be maintained during progression to MM, or be acquired later at MM. However, we found that acquisitions of driver mutations are subclonal in nature. Low variant allele frequencies were identified for RAS pathway mutations (*KRAS* range 0.024–0.53, *NRAS* 0.03–0.28), suggesting that these mutations were present in subclonal PC populations during progression (P01, P02, P05 and P06) (Fig. 1d). Interestingly, acquisition of *DIS3* mutations at the MM stage in patients P07 and P10 was observed to be clonal in nature [31] (Fig. 1d).

**Fig. 2:**
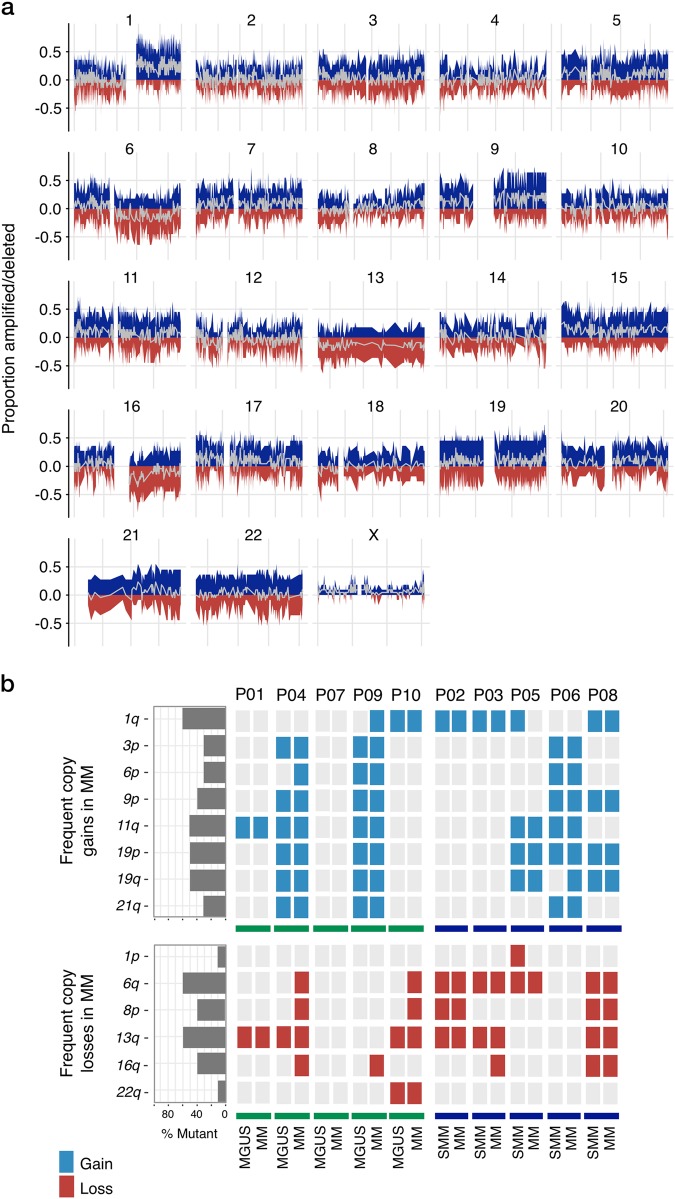
The chromosomal copy number variation landscape associated with MGUS/SMM to MM progression. **a** Chromosomal copy number landscape plot illustrating the proportion of patients with copy amplifications (blue) and deletions (red) of chromosomes across all patients associated with MGUS/SMM to MM progression. Grey traces the average in the cohort as a whole, where below 0 indicates loss and above 0 represents gain. **b** Frequent previously reported copy number changes of MM were identified, including gains on 1q, 3p, 6p, 9p, 11q, 19p, 19q and 21q; and losses on 6q, 8p, 13q, 16q and 22q being present at the asymptomatic stages and maintained with progression to MM

We also characterised the copy number variation (CNV) landscape associated with MGUS/SMM to MM progression, finding copy changes to be widespread (Fig. 2a). This contrasts a recent small longitudinal study of SMM to MM transformation that showed that copy changes are a feature of early stage of MM disease and not associated with progression [4]. We observed that MGUS/SMM patients harbour a similar frequency of chromosomal loci copy gains and losses than MM patients, with a median of 70 at MGUS/SMM (range 19 to 114), and 67.5 at MM (range 43 to 103) (Supplementary Table 5). Upon progression, we observed known frequent chromosomal copy number abnormalities in MM, including amplifications on chromosome arms 1q, 3p, 6p, 9p, 11q, 19p, 19q and 21q, coupled with losses on chromosome arms 1p, 6q, 8p, 13q, 16q and 22q across patients in the cohort (Fig. 2b). While we performed a gene level copy number analysis, we did not find any genes that were consistently gained or lost in our cohort upon progression. Interestingly, we also observed that many of the cytogenetic abnormalities associated with MM are present at MGUS/SMM stages and that standard cytogenetic methods did not accurately capture these abnormalities (Supplementary Figure 4).

### The subclonal architecture required for MM progression exists at MGUS/SMM diagnosis

While clonal heterogeneity is an established feature in MM, the subclonal evolution associated with disease progression has not been well explored. Due to the nature of our paired longitudinal patient samples, we were able to directly examine the relationship between genetic variegation and clonal structure to construct evolutionary trajectories accompanying progression to MM in eight individual patients.

Comparisons of unpaired MGUS/SMM and MM samples have shown that MGUS/SMM exhibits mutational similarity with MM, but many mutations were present in a smaller proportion of aberrant PCs [32, 33]. Similarly, small paired-sample studies examining the evolution over time of asymptomatic monoclonal gammopathies (AMGs) to MM (*n* = 4) [34], and high-risk SMM to MM patients (*n* = 4) [4], have also found that most somatic changes required for MM were present at the asymptomatic stages, with the clinically dominant MM subclone present at the SMM stage. Therefore, the occurrence of clonal evolution in MM represents a change in clonal heterogeneity over time from the asymptomatic stages to MM [13].

**Fig. 3:**
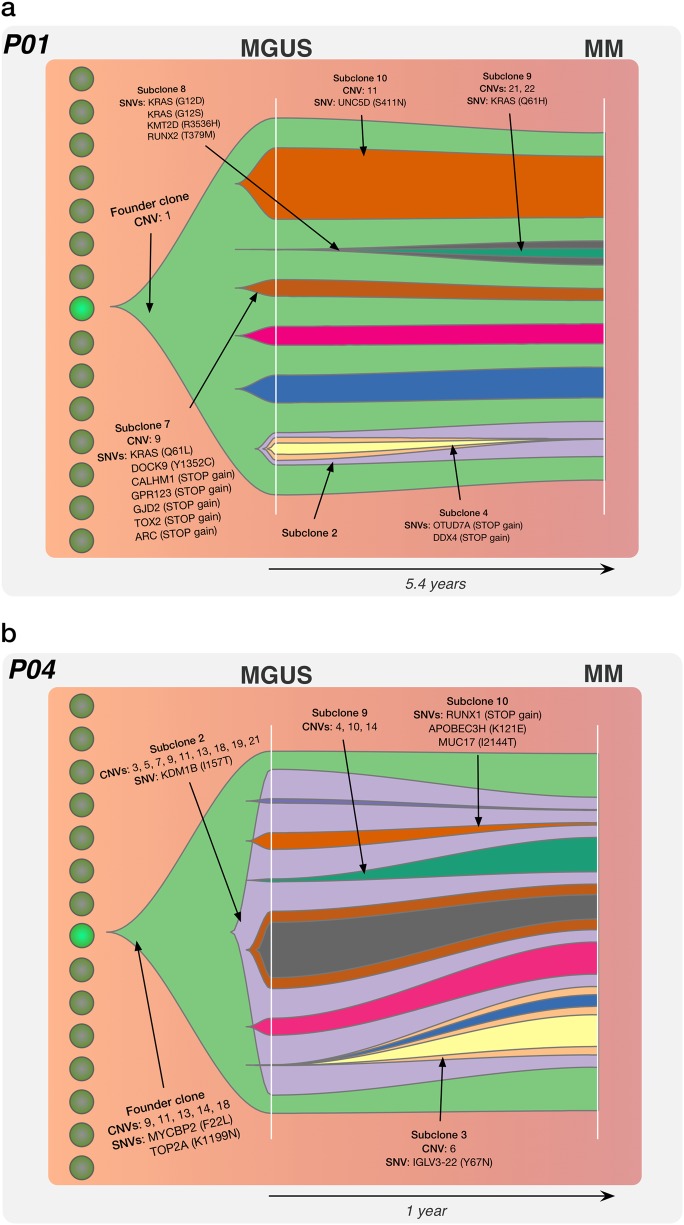
The subclonal tumour evolution associated with MGUS to MM progression. Fishtail plots illustrate the subclonal architecture in MGUS-MM of two patients (**a**: P01 and **b**: P04), which was defined by the existence of between 5 and 8 PC subclones at MGUS diagnosis. These subclonal populations generally progress to MM in a stable manner, in combination with the coupled emergence and/or extinction of child subclones. Key mutations in the founder clone and subclones are highlighted, with mutations in driver genes identified at both the clonal and subclonal level. The full-annotated subclonal genetic architecture for all patients can be found in Supplementary Figure 1

**Fig. 4:**
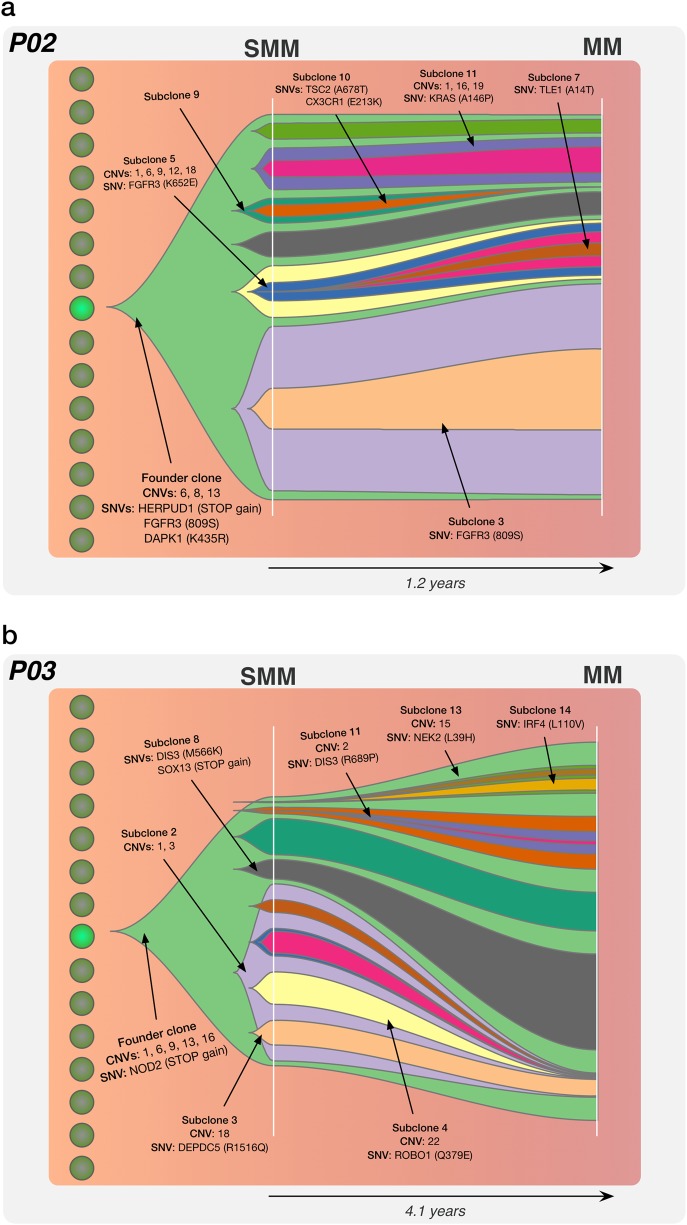
The subclonal tumour evolution associated with SMM to MM progression. Fishtail plots illustrating the subclonality in SMM-MM of two patients (**a**: P02 and **b**: P03), with the existence of between 5 and 11 PC subclones at the SMM stage. Notably, in comparison to the subclonal architecture at MGUS diagnosis, we observe a similar number of subclones present at SMM. Similar to the MGUS subclones, these SMM subclonal populations generally progress to MM in a stable manner, in combination with the coupled emergence and/or extinction of child subclones. Key mutations in MM genes in the founder clone and subclones are highlighted, where mutations in driver genes were found to be both clonal and subclonal in nature. The full-annotated subclonal genetic architecture for all patients can be found in Supplementary Figure 2

In both MGUS-MM (P01, P04 and P10) and SMM-MM (P02, P03, P05, P06 and P08) progression, we find a prevailing model of evolution defined by clonal stability. This is where the transformed subclonal PC populations identified at MM were already present in the asymptomatic MGUS/SMM stages. Progression to MM involved subtle changes in the existent subclonal structure from MGUS/SMM, coupled with a degree of emergence and/or extinction of child subclonal branches (Figs. 3 and 4). Of note, we observed that subclonal evolution has already begun prior to MGUS/SMM sampling. While multiple subclonal populations are present at MGUS/SMM diagnosis, each patient harbours unique set of oncogenic mutations driving MM progression.

#### Subclonal tumour evolution in MGUS-MM patients

Three patients (P01, P04 and P10) were initially diagnosed with MGUS, and subsequently with MM. Typically, an average of 7 subclones were identified in MGUS sample pairs. We describe two examples, with the full-annotated subclonal architecture for all MGUS-MM patients found in Supplementary Figure 1/Appendix 1.

Patient P01 exhibited a modest increase in NS-SNV mutations with progression and was composed of eight subclones at diagnosis. The founder clone had a copy number change on chromosome 1. Interestingly, while P01 mainly exhibited stable progression of subclones from MGUS to MM, we observed *KRAS* mutations to be newly acquired in multiple child subclones. Subclone 7 (brown) harboured a mutation causing an amino acid change at Q61L, with a resultant neutral growth observed. Furthermore, we identified mutations occurring in a nested fashion, with outgrowth of subclone 8 (grey from <1 to ~6%) harbouring mutations at G12D and G12S, with further emergence of child subclone 9 (green) harbouring additional change at Q61H with MM progression. This was coupled with the extinction of child subclonal branches of subclone 2 (purple) (Fig. 3a).

Patient P04 exhibited an interesting subclonal evolution pattern, where initially one subclone (subclone 2 purple) evolved from the founder clone, which was followed by substantial branching evolution resulting in six child subclones involved in MM progression. The founder clone harboured mutations in *MYCBP2* (F22L) and *TOP2A* (K1199N) and copy number changes on chromosomes 9, 11, 13, 14 and 18. While most of the child subclones exhibit stability, subclone 3 (orange from <1 to ~18%) and subclone 9 (green from <1 to ~9%) appear to have a selective advantage and showed emergence towards MM (Fig. 3b). Similarly, P10 was composed of eight subclones at MGUS with the neutral growth of subclonal populations coupled with the emergence of multiple subclones (subclone 4 yellow from ~3 to ~25%, and subclone 5 blue from ~1 to ~10%) and extinction of child subclone 3 (orange from ~5 to <1%) with progression (Supplementary Figure 1c).

#### Subclonal tumour evolution in SMM-MM patients

Five patients (P02, P03, P05, P06 and P08) were diagnosed for SMM, and then subsequently MM at a later time point. Generally, an average of 8 subclones were identified in SMM-MM pairs. We report two examples, with the full-annotated subclonal architecture for all SMM-MM patients found in Supplementary Figure 2/Appendix 1.

Patient P02 was composed of eleven subclones at diagnosis and exhibited stable growth during progression with mainly the emergence of child subclone 5 and its branches (blue from ~5 to ~13%) and extinction of subclone 9 (dark green from ~6 to <1%) (Fig. 4a). The founder clone showed copy changes on chromosomes 6, 8 and 13, and mutations in *HERPUD1* (STOP gain), *FGFR3* (809S) and *DAPK1* (K435R). Furthermore, we identified a *KRAS* mutation (A146P) in subclone 11, whose population proportion size, interestingly, did not change during MM progression.

Patient P03 displayed an interesting evolution pattern with massive extinction of subclone 2 (purple) from ~47 to ~6%, and almost all of its child subclones, by MM diagnosis. The founder clone harboured mutations in *NOD2* (STOP gain) and CNVs on chromosomes 1, 6, 9, 13 and 16. Furthermore, two individual subclones that contained distinct *DIS3* mutants M566K and R689P were identified at SMM diagnosis in subclone 8 (black) and child subclone 11 (dark purple), respectively (Fig. 4b). While recent single-cell analysis has demonstrated parallel evolution of the RAS/MAPK pathway in MM through the occurrence of *RAS* mutations in individual clones leading to distinct subclonal populations [35], here we uniquely identify parallel evolution of *DIS3*, with the resultant emergence of both subclonal lineages with MM progression. Additionally, subclone 13 and its child subclones exhibited outgrowth with a mutation in *NEK2* (L39H) (light green from <1 to ~7%).

Clonal stability in tumour evolution is also exemplified in other SMM-MM patients (P05, P06 and P08), which were characterised by 7, 5 and 8 subclones at diagnosis, respectively, and exhibited coupled emergence and extinction of child subclones in the progression to MM. In P05, there were initially two subclones that progressed to MM with the emergence and extinction of child clones from subclonal branch 5 (blue), combined with the neutral growth from subclonal branch 2 (purple) (Supplementary Figure 2c). Similarly, in P06, with progression we observed the emergence of subclone 8 (black from <1 to ~23%) and its child subclone 9 (dark green from <1 to ~4%), and subclone 2 (purple from ~8 to ~28%) and its child subclones 6 (pink from <1 to ~10%) and 7 (brown from <1 to ~6%). The proportions of child subclonal population 3 (orange) remained unchanged between SMM and MM (Supplementary Figure 2d). Patient P08 exhibited neutral growth, which was coupled with the emergence of child subclone 9 (green from <1 to ~5%) and extinction of child subclone 8 (black from ~6 to <1%) with MM progression (Supplementary Figure 2e).

Our analysis reveals conclusive evidence of intraclonal heterogeneity and subclonality from the earliest MGUS/SMM stages, where most of the transformed subclonal populations involved in progression to MM are already present at diagnosis. Notably, we do not observe a remarkable difference in the subclonality characteristic at the initial asymptomatic MGUS stage (average 7 subclones) and the intermediate SMM stage (average 8 subclones). This suggests that major subclonal remodelling is also not a phenomenon associated with advancement between the asymptomatic stages.

## Discussion

The longitudinal investigation of MGUS/SMM to MM samples using NGS has revealed a new understanding of the underlying genetic architecture and subclonal evolution associated with MM progression. Analysis of MGUS-MM, and SMM-MM transition has shown that intraclonal heterogeneity is present at the asymptomatic stages. We find that progression is associated with an altered landscape of acquired mutations, rather than an increased total mutational burden.

Cancer progression models propose either the sequential accumulation of key genetic mutations throughout progressive disease and clonal expansions (‘Darwinian’ evolution), or punctuated bursts of large-scale chromosomal alterations (‘Saltationist’ evolution) [36]. The current understanding of MM transformation involves a sequential nature of evolution from the well-defined asymptomatic stages of MGUS and SMM, characterised by clonal expansion of PCs, and branching ‘Darwinian’ evolution with the presence of 2 to 6 subclones, highlighting clonal heterogeneity at MM presentation [10, 18–20, 29, 30, 35, 37–41]. In this model it is recognised that progression from the asymptomatic stages is dependent on the rise and fall in dominance of PC subclones based on their clonal fitness. The acquisition of driver mutations confers a selective advantage and facilitates better survival properties allowing the subclones to survive the selective pressures of the microenvironment/immune system and progress to symptomatic MM.

Notably, our study establishes MM disease progression to be characterised by the phenomenon of clonal stability, where substantial remoulding of the subclonal populations from the asymptomatic stages is not a necessary prerequisite for progression to MM. We found the existence of multiple PC subclones (range 5–11) at both MGUS and SMM that were intrinsic in the development and progression of MM. Furthermore, by comparing patients at MGUS and SMM stages we identified no significant difference in the number of PC subclones present at diagnosis (with an average of 7 versus 8, respectively). This is striking, as progression between the asymptomatic stages of MGUS and SMM is currently distinguished by an increased BM PC% and monoclonal protein level. We also found no correlation between the extent of subclonality and BM PC% at the MM stage in patients (Supplementary Figure 3). Similarly, a recent study of four high-risk SMM to MM transformation patients revealed that clonal progression was the key feature of MM onset, where the invasive clinically predominant clone typical of MM, was already present at SMM [4]. Similarly, in their investigation, Walker et al. reported a shifting clonal structure with the outgrowth and reduction of subclonal populations from SMM to MM [4]. Taken together, we hypothesise that patients who progress within a short time frame, MGUS/SMM to MM transformation does not always required the acquisition of many additional mutations and clonal selection. These MGUS/SMM patients appear to be sufficiently genetically complex to be on the threshold of transformation to MM, which may possibly be driven by extrinsic factors.

Being able to define the crucial oncogenic events in the founder clonal population could facilitate treatment strategies for early intervention to arrest MM progression. However, as the subclones responsible for MM are evident at the asymptomatic stages this poses the question as to why these patients are not symptomatic. A strong possibility is that further to intrinsic genetic factors, extrinsic factors such as the tumour microenvironment may also play an important role in defining both the subclonal architecture and the overall tumour cell burden for progression to clinical malignancy. The complex interactions of the tumour microenvironment with subclones provide signals that may support tumour growth or dormancy, which may influence their transformation [42–49]. Of note, a recent study from our group which used the C57BL/KaLwRij mouse model of MM, demonstrated habitual clonal dominance, where only a few establishing MM cells subsequently contributed to tumour burden while most remained dormant. This illustrates the strong selection pressures present within the BM microenvironment which plays a role in defining the clonal architecture [46].

The current standard of care at the asymptomatic stages involves monitoring patients, with no treatment options until they progress to symptomatic MM. Here our study has demonstrated that there is no significant shift in subclonal structure associated with MM progression. As such, subclonal populations present at MGUS/SMM diagnosis would be just as amenable to treatment, and eradication of these subclonal populations prior to disease transformation could delay progression and may provide the prospect of a durable cure [50]. However, we recognise that intraclonal heterogeneity has been shown to be characteristic of MGUS/SMM/MM, with multiple subclones having differing survival properties, therefore the risk of further mutation and tumour evolution due to drug selection pressures would eventually lead to relapse. Furthermore, intraclonal heterogeneity with clonal selection may not be the only defining evolution associated with progression of MM, with a recent study illustrating the involvement of spatial heterogeneity with regional site seeding and outgrowth resulting in progression [51]. Therefore, a combined longitudinal and spatial study of progression in patients would further elucidate genomic biomarkers of MM tumour evolution [52], although the ability to sample from multiple sites in asymptomatic patients has significant ethical and practical challenges.

Our findings reveal new insights into the genomic complexity and subclonal tumour evolution that is present from MGUS/SMM through to MM transformation. The existence of subclonality and clonal stability as a model of tumour evolution not only provides a more comprehensive understanding of the underlying biology of MM disease progression, but also new considerations required for patients at diagnosis and future therapeutic approaches to control this disease.

## Electronic supplementary material

Leukemia_Dutta_Supplementary Final File

Supplementary Figure 1

Supplementary Figure 2a

Supplementary Figure 2b

Supplementary Figure 2c

Supplementary Figure 3a

Supplementary Figure 3b

Supplementary Figure 3c

Supplementary Figure 3d

Supplementary Figure 3e

Supplementary Figure 4

Supplementary Figure 5
