# Supplementary material for: Subclonal evolution in disease progression from MGUS/SMM to multiple myeloma is characterised by clonal stability
Source: Leukemia. 2018 Jul 25;33(2):457–68. doi: 10.1038/s41375-018-0206-x (PMC6365384; doi:10.1038/s41375-018-0206-x)
Supplement: Supplementary file 1 — Leukemia_Dutta_Supplementary Final File [file 41375_2018_206_MOESM1_ESM.docx]

***Leukemia, Article Supplementary***

***Subclonal evolution in disease progression from MGUS/SMM to multiple myeloma is characterised by clonal stability***

Ankit K. Dutta^1,3^, J. Lynn Fink^2*^, John P. Grady^2^, Gareth J. Morgan^4^, Charles G. Mullighan^5^, Luen B. To^6,7^, Duncan R. Hewett^1,3^ & Andrew C.W. Zannettino^1,3*^

^1^Myeloma Research Laboratory, Adelaide Medical School, Faculty of Health and Medical Sciences, The University of Adelaide, Adelaide, SA, 5005, Australia.

^2^Genomic Medicine Division, The University of Queensland, Diamantina Institute (UQDI), Brisbane, QLD, 4102, Australia.

^3^Cancer Theme, South Australian Health and Medical Research Institute (SAHMRI), Adelaide, SA, 5000, Australia.

^4^The Myeloma Institute, University of Arkansas for Medical Sciences, Little Rock, AR, 72205, USA.

^5^Department of Pathology and the Hematological Malignancies Program, St Jude Children’s Research Hospital, Memphis, TN, 38105, USA.

^6^SA Pathology, Adelaide, SA, 5000, Australia.

^7^Haematology and Bone Marrow Transplant Unit, Royal Adelaide Hospital, Adelaide, SA, 5000, Australia.

*co-senior authors

***Supplementary Methods***

**Whole Exome Sequencing.**

Exome libraries were generated using the Nimblegen KAPA Hyper Library Prep kit (Kapa Biosystems, PN KP-KK8504) followed by the SureSelectXT Clinical Research Exome (CRE) (Agilent, S06588914) capture kit. 115ng of gDNA were used as input for fragmentation on the Covaris E220 followed End-Repair/A-Tailing and ligation of the SureSelect Adapter Oligos, excepting samples which had low input and required additional PCR cycles [*Supplementary Table 6*]. 10 cycles, or 12 cycles in the case low input samples, of Pre-Capture PCR amplification were performed to produce sufficient library for exome capture. Libraries were quantified on the LabChip GX II (LCGXII) using the 5K HT DNA assay (Perkin Elmer, PN 760435 and CLS760675) and 750ng of each sample was input to the Agilent CRE capture workflow, hybridised to the CRE probes overnight. Following the capture washes and 11 cycles of Post-Capture PCR incorporating index barcodes, captured libraries were validated on the LCGXII using the 5K HT DNA assay (Perkin Elmer, PN 760435 and CLS760675). An equimolar pool was prepared from the captured libraries and the pool validated on an Agilent HS DNA Bioanalyzer chip (Agilent, PN 5067-4626), and by qPCR using the KAPA Library Quantification Kits (Kapa Biosystems, PN KK4824), to assess quantity and quality of the samples ready for sequencing. Sequencing was performed on the Illumina HiSeq4000 (2x100 bp paired-end reads) and NextSeq 500 (2x150 bp paired-end reads). Samples were sequenced to a minimum depth of ~140X mean coverage. Isolated non-tumour cells were also sequenced to a similar average depth (138x).

**Analysis of Whole Exome Sequencing Data.**

*Sequence alignment.*

Sequencing reads were mapped to the human decoy genome (hs37d5) using Novoalign (v3.02.08) followed by post-processing according to GATK best practices^21^.

*Somatic variant calling.*

Somatic single nucleotide and small indel variants were called using MuTect2^22^ and multiSNV^23^. Variants were filtered using the following criteria: 10+ reads covering the variant site; 5+ reads covering the variant in the tumour sample. Variant annotation was performed with SnpEff^24^.

R 3.3.2 was used throughout for analyses. Somatic copy number variants were called using CNVkit^25^ v0.7.11 and custom in-house methods developed to support highly aneuploid genomes to perform segmentation and calculate log2 change between matched non-tumour and MGUS/SMM/MM. Log2 changes were corrected for sample purity to calculate ploidy at each stage. To investigate common copy number changes between MGUS/SMM and MM stages, for each patient and each gene the ploidy change was calculated between MGUS/SMM and MM, and a score generated for each gene by calculating the number of patients demonstrating ploidy increase minus the number of patients demonstrating ploidy decrease (ploidy change was threshold at 0.2 copies to reduce noise). Broad copy number changes were compared to cytogenetics data when available to examine concordance. Focal copy number changes were defined as regions < 3Mb in length^26^

To investigate total copy number change at each stage, the CNVkit segmentation was used. Purity adjusted ploidy changes > 0.2 were summed for each patient. The Y chromosome, Immunoglobulin heavy region on chromosome 14, and T cell receptor A variable regions on chromosomes 7 and 14 were excluded from this analysis as they were frequently hyper-segmented in the CNVkit analysis.

**Tumour heterogeneity and subclonal evolution.**

Clonal evolution was investigated using PhyloWGS^27^ and visualised using fish plot in R^28^. It is worth noting that PhyloWGS can inflate the number of subclones so, although we based our analysis on the inferred subclonal architecture, we recognise that the numbers of subclones may be overestimated. There are some mutation discrepancies between the subclonal trees and the SNVs called by MuTect2 due to threshold differences between the two analyses. PhyloWGS requires one time point to demonstrate that the mutation is present at a non trivial level, therefore a lower burden of proof is required to infer its existence between the two time points. However, if both time points demonstrate a low proportion then that mutant is not inferred in the subclonal tree. In our subclonal tumour evolution models, we did not consider polyclonal evolution, where multiple founder PC clones were present at the MGUS/SMM diagnosis stage, due to the computational difficulty in modeling polyclonal evolution. All phylogenetic trees constructed were based on the assumption that there is a single founder PC clone.

***Supplementary Appendix 1***

*Subclonal tumour evolution in MGUS-MM patients*

Patients P01, P04 and P10 were initially diagnosed with MGUS, and subsequently with MM. We observed that MGUS-MM patients exhibited an average time to progression (TTP) of ~5 years. All three patients showed a decrease in total non-synonymous SNVs associated with progression [*Figure 1a*]. Patients P04 and P10 were composed of eight and five subclones at MGUS diagnosis, respectively. Patient P04 exhibited an interesting subclonal evolution pattern, where initially one subclone [subclone 2 purple] evolved from the founder clone, which was followed by substantial branching evolution resulting in six child subclones involved in MM progression, and a rapid TTP of 1 year. The founder clone harboured mutations in *MYCBP2* (F22L) and *TOP2A* (K1199N) and copy number changes on chromosomes 9, 11, 13, 14 and 18. While most of the child subclones exhibit stability, subclone 3 [orange] and subclone 9 [green] appear to have a selective advantage and showed emergence towards MM [*Supplementary Figure 1b*].

In patient P10 we observed a decreased proportion of the founder clone, likely due to some normal PCs contamination [*Supplementary Table 2*]. We identified neutral growth of subclonal populations coupled with the emergence of multiple subclones [subclone 4 yellow from ~3% to ~25%, and subclone 5 blue from ~1% to ~10%] and extinction of child subclone 3 [orange from ~5% to <1%] with progression. The founder clone showed multiple high impact mutations in *DUSP27* (STOP gain), *SP140* (F133I) and *FAM110B* (P339L) [*Supplementary* *Figure 1c*]. The TTP of P10 was noted to be 13 years, possibly representing an earlier diagnosis and sampling time for this patient.

Patient P01 exhibited a slight decrease in NS-SNV mutations with progression and was composed of eight subclones at diagnosis. The founder clone had a copy number change on chromosome 1. Interestingly, while P01 mainly exhibited stable progression of subclones from MGUS to MM, we observed *KRAS* mutations to be newly acquired in multiple child subclones. Subclone 7 [brown] harboured a mutation causing an amino acid change at position Q61L, with a resultant neutral growth observed. Furthermore, we identified mutations occurring in a nested fashion, with outgrowth of subclone 8 [grey from <1% to ~6%] harbouring mutations at G12D and G12S, with further emergence of child subclone 9 [green] harbouring additional change at Q61H with MM progression. This was coupled with the extinction of child subclonal branches of subclone 2 [purple] [*Supplementary Figure 1a*].

*Subclonal tumour evolution in SMM-MM patients*

Patients P02, P03, P05, P06 and P08 were diagnosed for SMM, and then subsequently MM at a later time point. SMM patients demonstrated an average TPP of 2 years. Patients were separated on the basis of total non-synonymous SNV burden associated with MM progression, where 3 patients showed a decrease (P02, P05 and P08) and 2 patients showed an increase (P03 and P06) [*Figure 1a*]. Patients P02 and P06 were composed of eleven and five subclones at diagnosis, respectively. Patient P02 exhibited stable growth during progression, with mainly the emergence of child subclone 5 and its branches [blue from ~5% to ~13%] and extinction of subclone 9 [dark green from ~6% to <1%] [*Supplementary Figure* 2a]. The founder clone showed copy changes on chromosomes 6, 8 and 13, and mutations in *HERPUD1* (STOP gain), *FGFR3* (809S) and *DAPK1* (K435R). Furthermore, we identified a *KRAS* mutation (A146P) in subclone 11, whose population proportion size, interestingly, did not change during MM progression.

Patient P06 displayed a small founder clone proportion, possibly due to variants such as structural changes unable to be characterised by WES, which harboured copy changes on chromosomes 3, 5, 6, 15, 17, 19 and 21, and point mutation in *NRAS* (Q61R). We mainly observed the emergence of subclone 8 [black from <1% to ~23%] with *KLC3* mutation (R442H) and its child subclone 9 [dark green from <1% to ~4%], and subclone 2 [purple from ~8% to ~28%] and its child subclones 6 [pink from <1% to ~10%] with mutations in *MYCBP2* (E730K), *FGFR3* (A165T) and *PRDM1* (G214R) and 7 [brown from <1% to ~6%] with progression. The proportions of child subclonal population 3 [orange] remained unchanged between SMM and MM [*Supplementary Figure 2d*].

Patient P08 exhibited neutral growth, which was coupled with the emergence of child subclone 9 [green from <1% to ~5%] and extinction of child subclone 8 [black from ~6% to <1%] with MM progression. The founder clone had widespread mutations with CNVs in chromosome 2, 8, 9, 13, 16, 18, 19, 20 and 22, and SNVs in *RB1* (G449E), *PLEKHA7* (STOP gain), *RBM4B* (STOP gain), *DDX55* (R222Q), *CCDC105* (STOP gain), *HIST1H3J* (STOP gain) and *MLIP* (STOP gain) [*Supplementary Figure 2e*].

Similar to P06, patient P05 showed a smaller founder clone proportion at diagnosis. There were initially two subclones present at the SMM stage, which progressed to MM with the emergence and extinction of child clones from subclonal branch 5 [blue], combined with the neutral growth from subclonal branch 2 [purple]. Subclone branch 5 [blue] and its child subclonal branches 9 [green] and 10 [light brown] harboured multiple stop mutations in genes *NRG3*, *EZH2*, *KLHL20*, *SNX9*, *C8orf87*, *ACTL6A* and *MTA3*. However, these child subclones became progressively extinct with MM progression, from ~5% to ~2% and ~3% to <1%, respectively. While child subclones 7 [dark brown] and 8 [black] exhibit emergence towards MM, from <1% to ~10% and <1% to ~5%, respectively. The founder clone harboured mutations in *KRAS* (G12V) and *ICAM5* (R85L), and CNVs on chromosome 2 and 19 [*Supplementary Figure 2c*].

Patient P03 displayed an interesting evolution pattern with massive extinction of subclone 2 [purple] from ~47% to ~6%, and almost all of its child subclones, by MM diagnosis. The founder clone harboured mutations in *NOD2* (STOP gain) and CNVs on chromosomes 1, 6, 9, 13 and 16. Furthermore, two individual subclones that contained distinct *DIS3* mutants M566K and R689P were identified at SMM diagnosis in subclone 8 [black] and child subclone 11 [dark purple], respectively [*Supplementary Figure 2b*]. While recent single cell analysis has demonstrated parallel evolution of the RAS/MAPK pathway in MM through the occurrence of RAS mutations in individual clones leading to distinct subclonal populations24, here we uniquely identify parallel evolution of *DIS3*, with the resultant emergence of both subclonal lineages with MM progression. Additionally, subclone 13 and its child subclones exhibited outgrowth with a mutation in *NEK2* (L39H) [light green from <1% to ~7%].

***Supplementary Figures***

******

**Supplementary Table 1. Clinical cytogenetic data for MGUS/SMM to MM patients.** Clinically recorded data at MM diagnosis for patients in the study. The median age of patients at MM diagnosis was 75.5 years. Molecular cytogenetics of patients was performed using FISH analysis on interphase spreads of bone marrow smears. Nil represents parameter not being present. N/A represents that data was not available.

**Supplementary Table 2. Estimated sample purity and exome sequencing coverage.** Purity of FACS sorted patient PCs was assessed by FACS purity check post sort on sorted cells tube, with 100-500 cells through the flow cytometer for each sample. Mean depth of sequencing describing the average number of reads over bases in the targeted exome region of samples sequenced on the HiSeq4000 and NextSeq500.

**Supplementary Table 3. The shared NS-SNVs between MGUS/SMM and MM, and unique NS-SNVs in MM patients.** Analysis of the total mutational load reveals a median of 161 NS-SNVs at MGUS/SMM and 152 NS-SNVs at MM. The table describes the shared and MM unique NS-SNVs identified in each patient.

**Supplementary Table 4. The full characterisation of driver mutations in MGUS/SMM to MM patients.** Single nucleotide variants in previously reported driver genes were identified in *KRAS*, *NRAS* and *DIS3*. The table describes the genomic positions and subsequent impact on cDNA and amino acid changes.

**Supplementary Table 5. The copy number landscape of MGUS/SMM to MM patients.** We identified numerous CNV changes in each patient at MGUS/SMM and MM. MGUS/SMM patients harboured a higher median number of changes than at MM (70 vs. 67.5, respectively)**.**

**Supplementary Table 6. Exome library preparation of low input samples requiring additional PCR cycles.** Two samples had yields lower than the required 115ng gDNA input and required extra PCR amplification during library preparation to generate sufficient library for exome capture.

**a b**

**
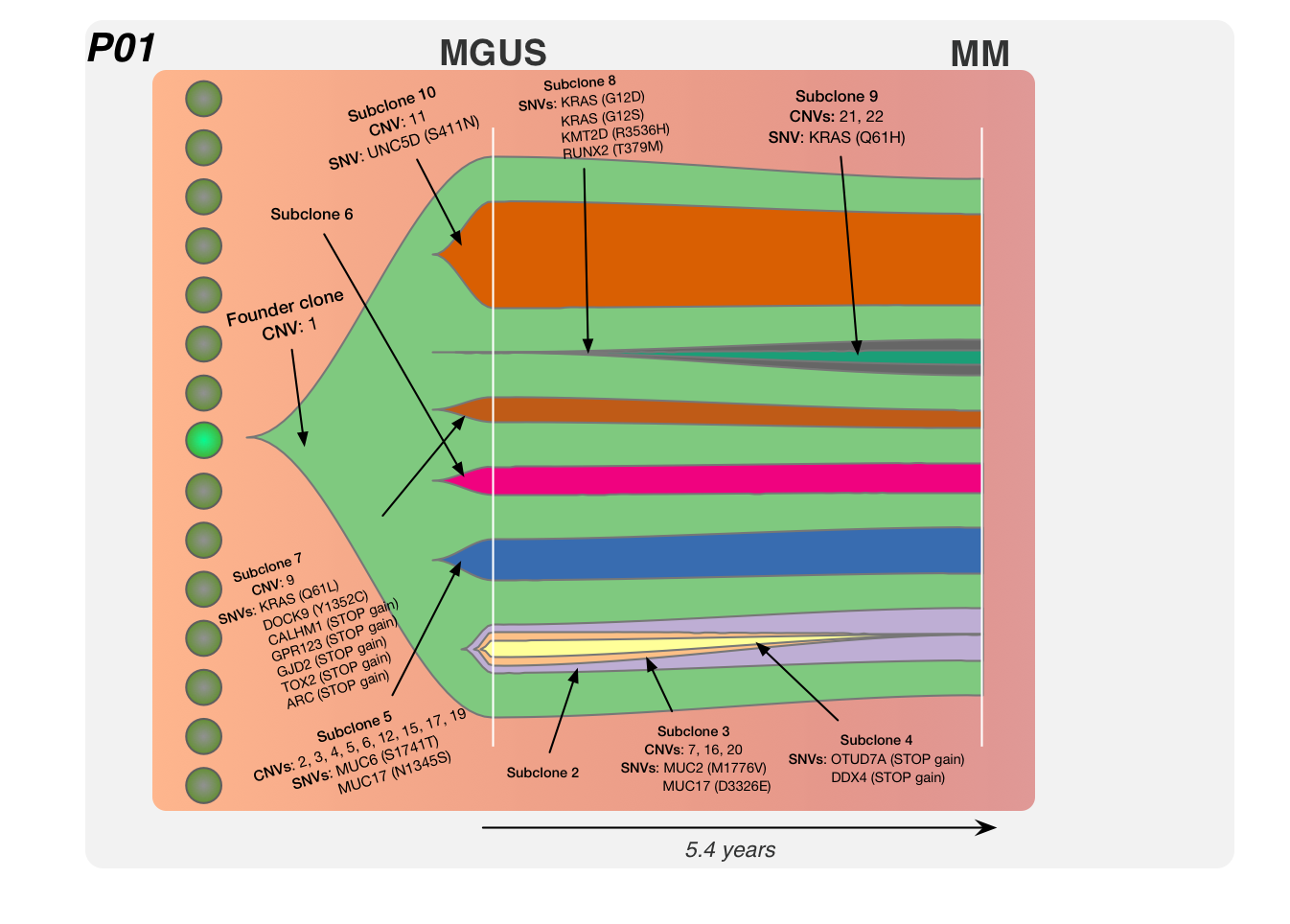

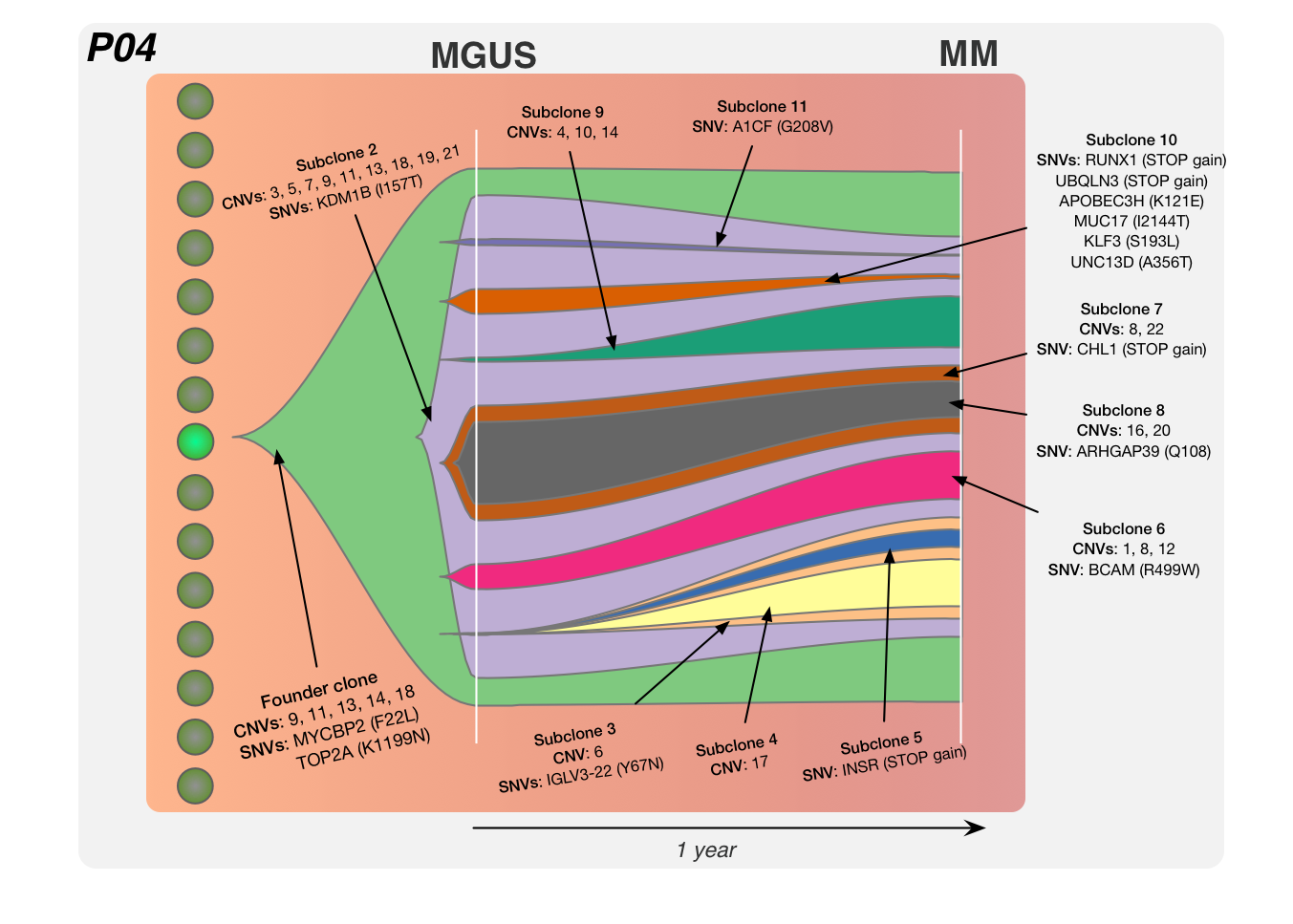
**

**c**

**
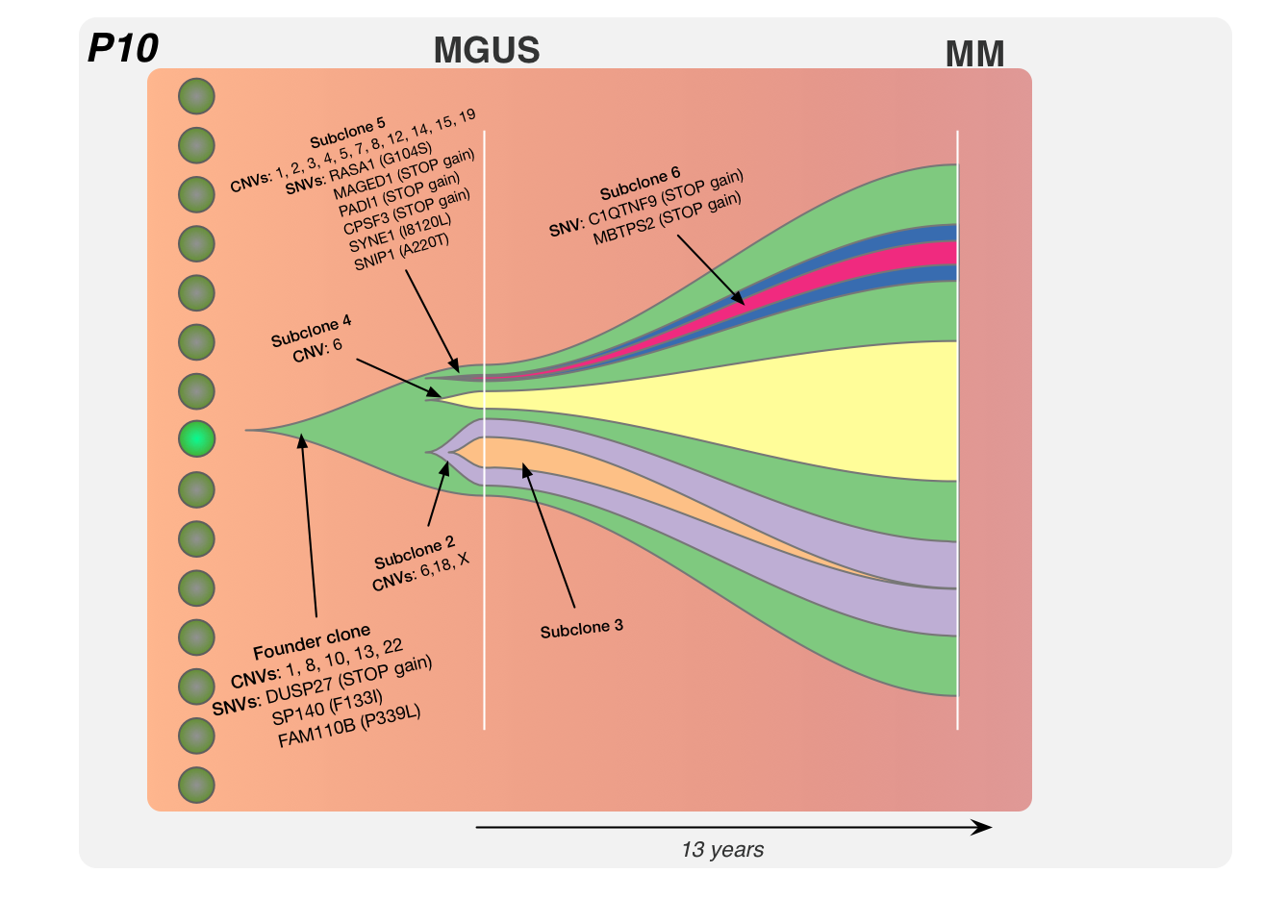
**

**Supplementary Figure 1.** **The subclonal tumour evolution associated with MGUS to MM progression.** Fishtail plots annotated with the complete subclonal genetic architecture in three patients (**a**: P01, **b**: P04 and **c**: P10) from Figure 3 of the main article.

**a b**

**
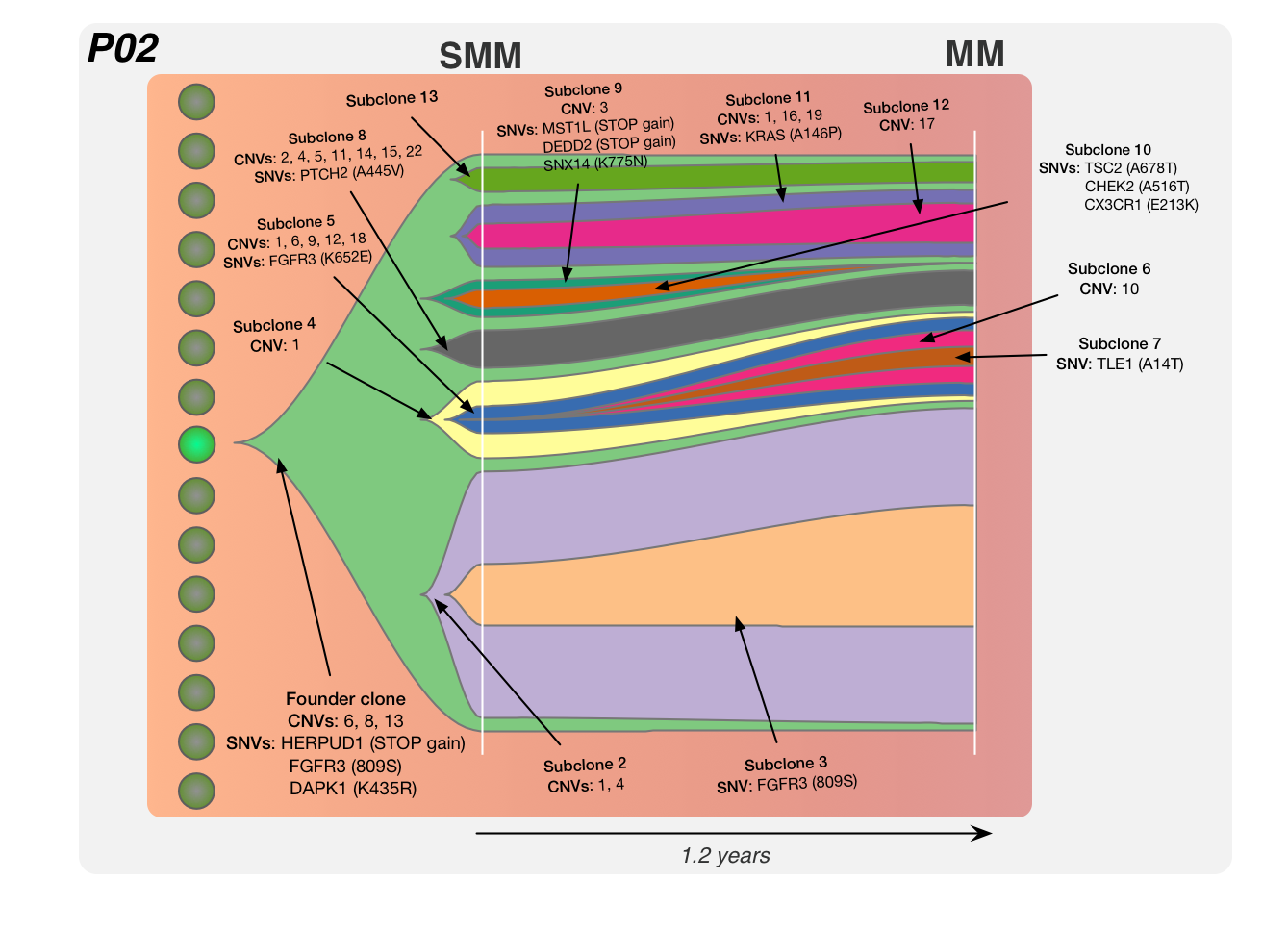

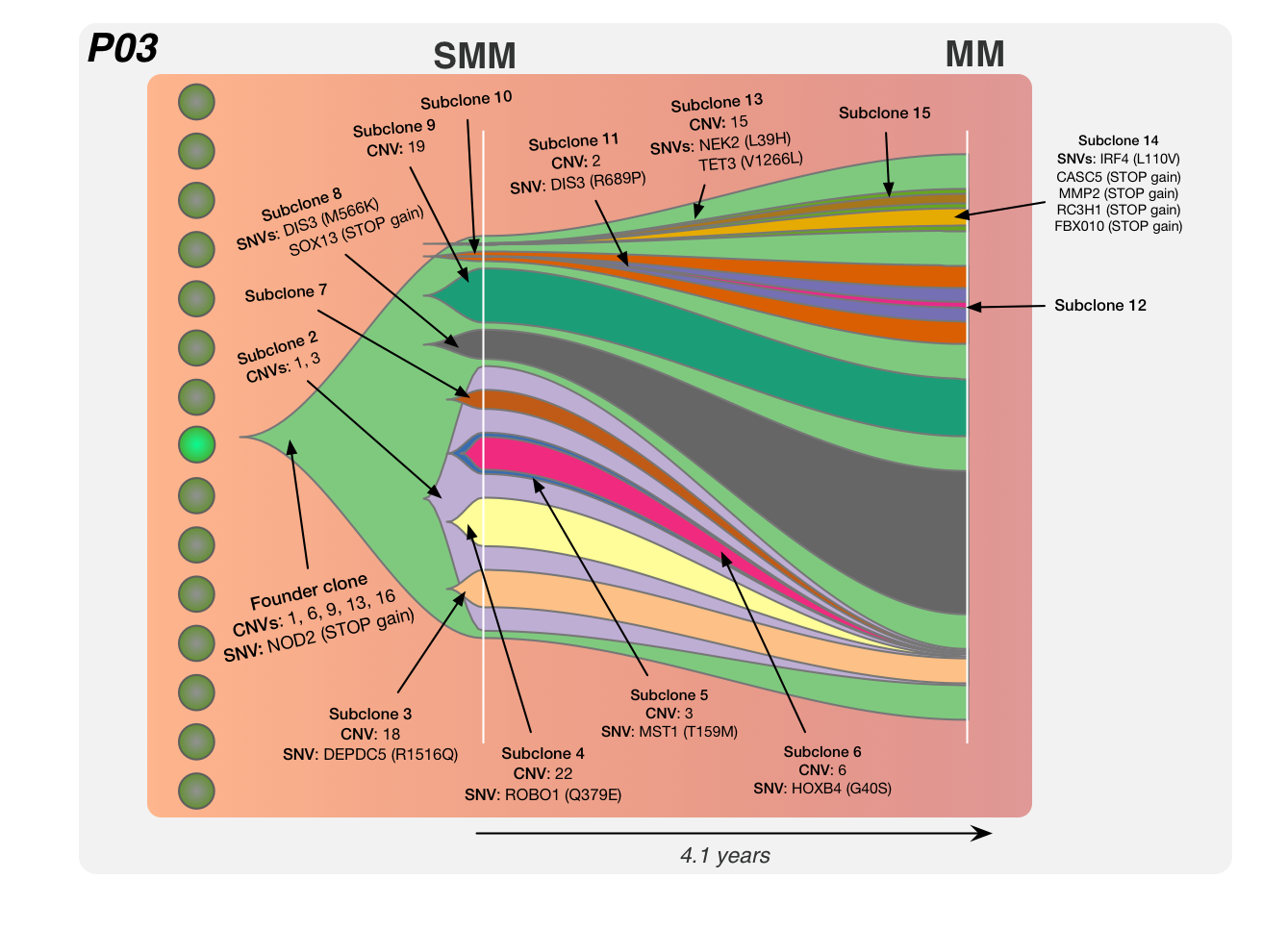
**

**c d**

**
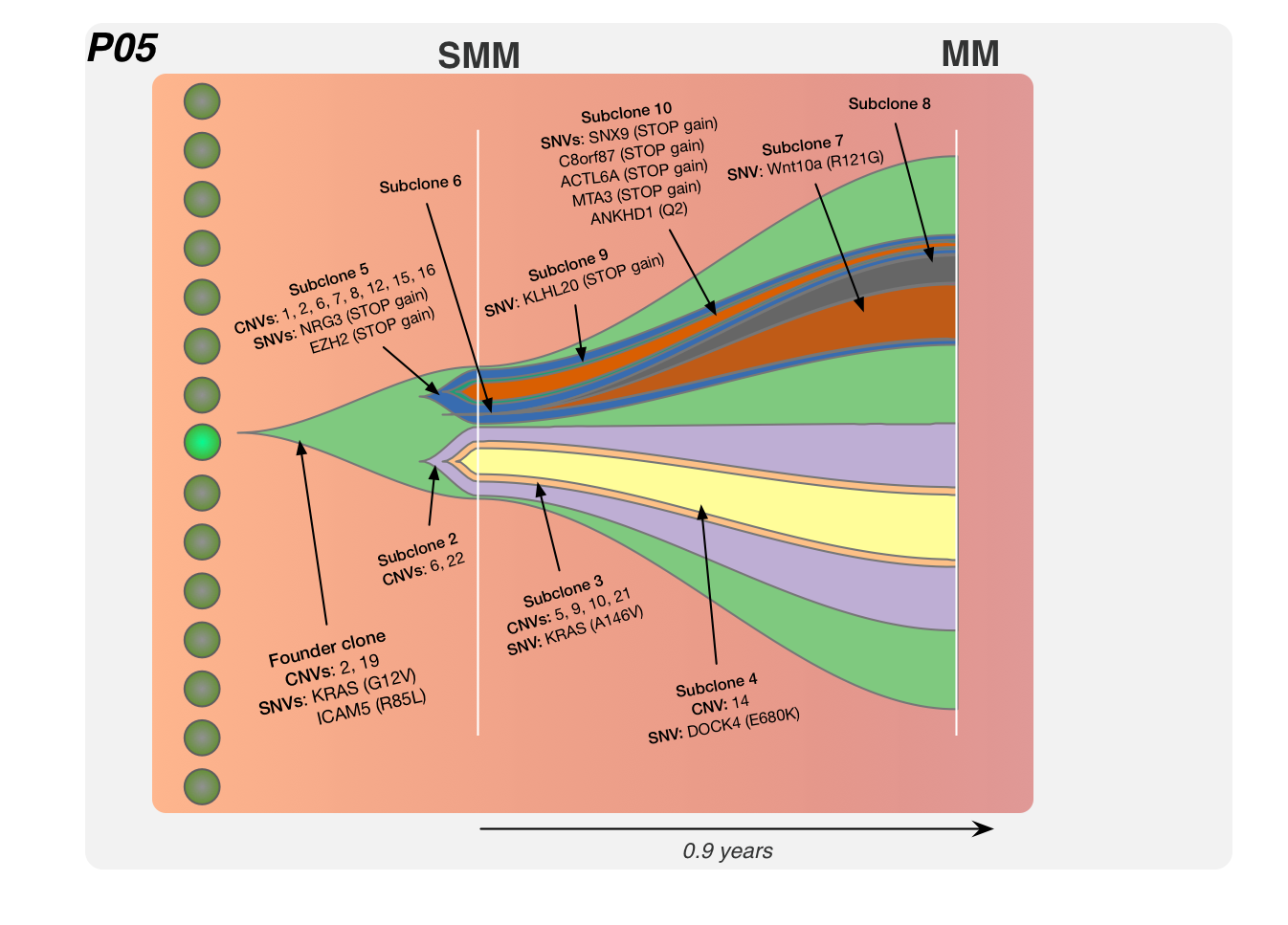

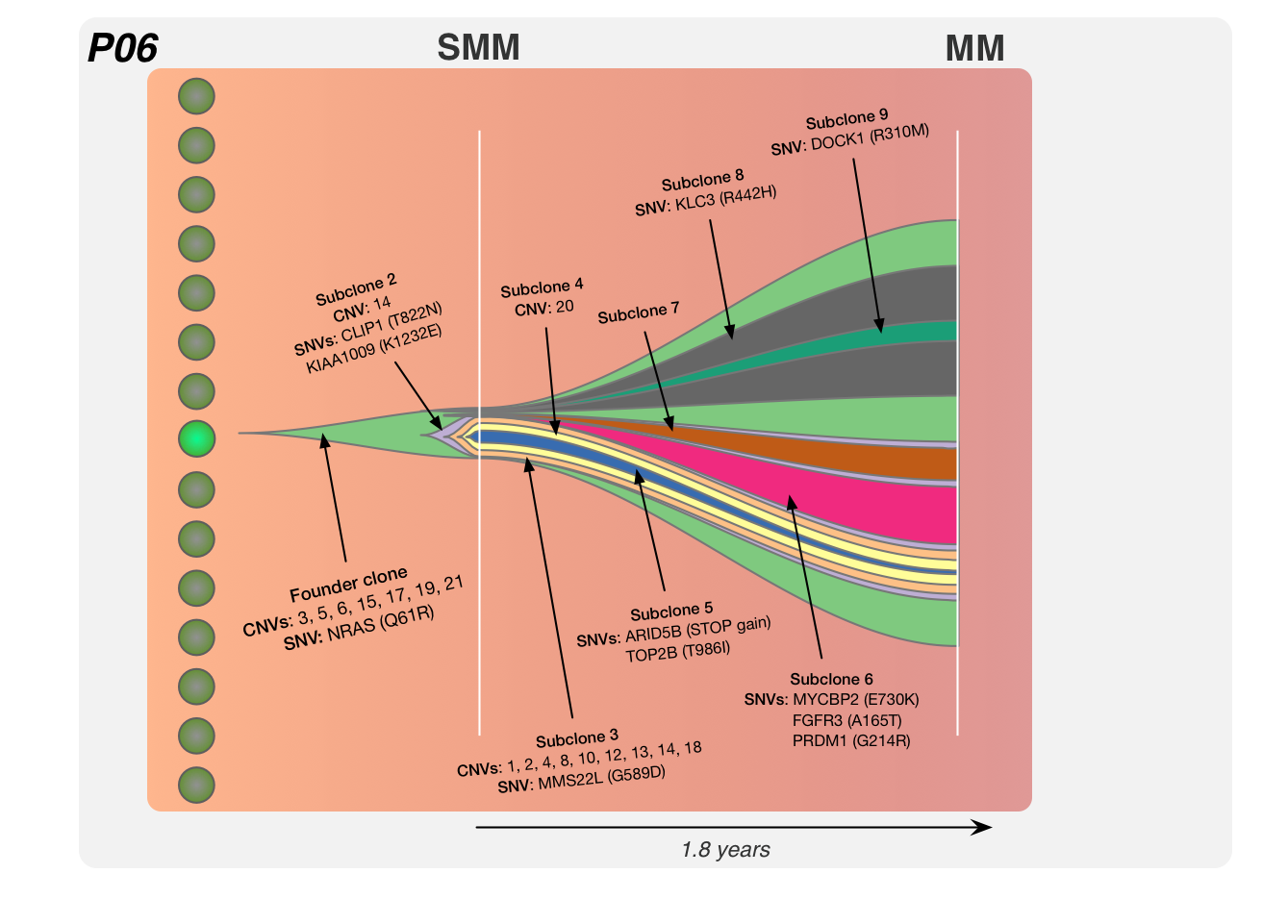
**

**e**

**
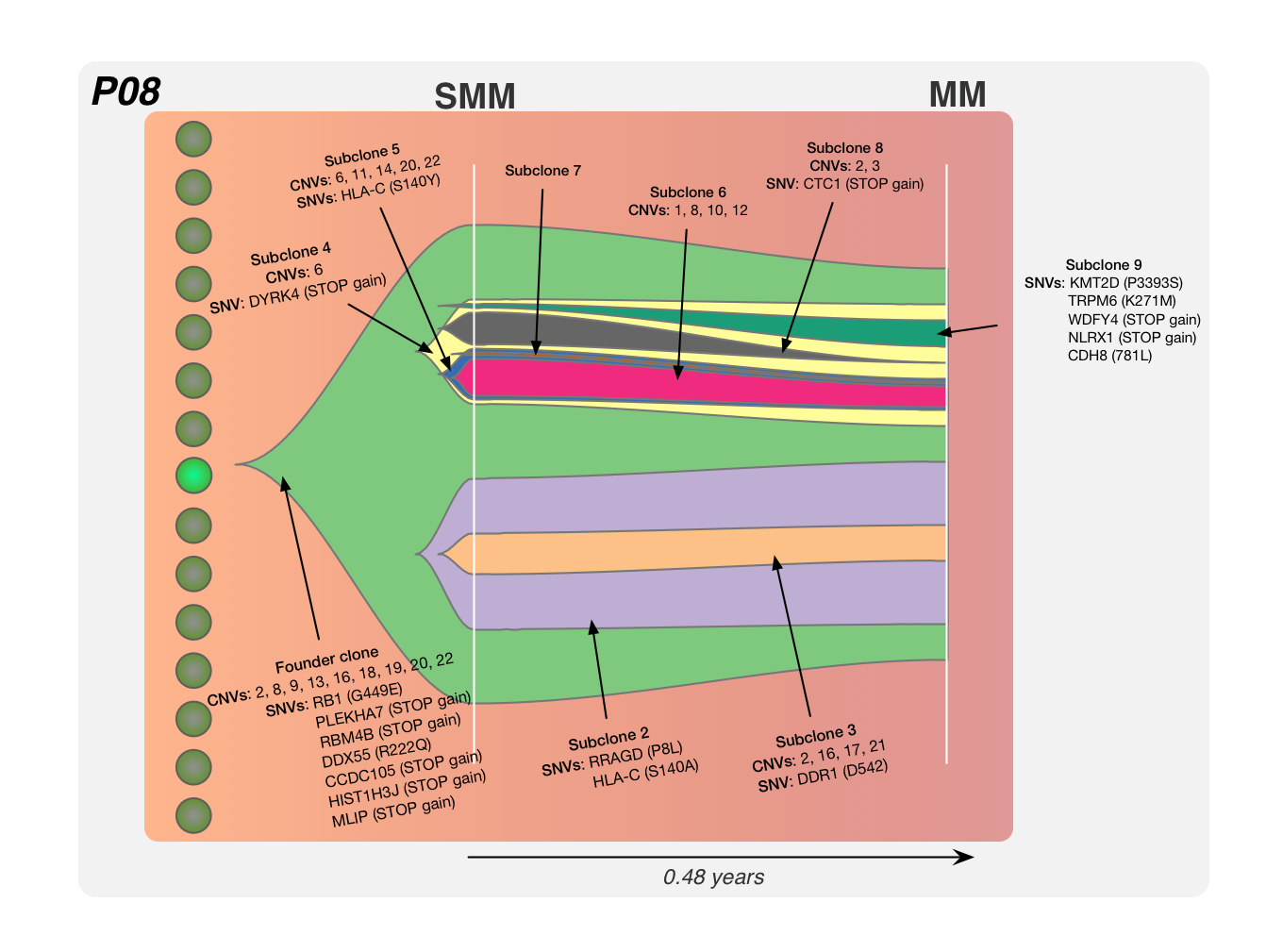
**

**Supplementary Figure 2.** **The subclonal tumour evolution associated with SMM to MM progression.** Fishtail plots annotated with the complete subclonal genetic architecture in five patients (**a**: P02, **b**: P03, **c**: P05, **d**: P06 and **e**: P08) from Figure 4 of the main article.

**Supplementary Figure 3. The correlation of BM PC% and the subclones identified with progression in MGUS/SMM to MM patients.** Progression to MM is characterised by an increase in BM PC% and monoclonal protein levels, however, we find no correlation between the extent of subclonality and BM PC% at MM.


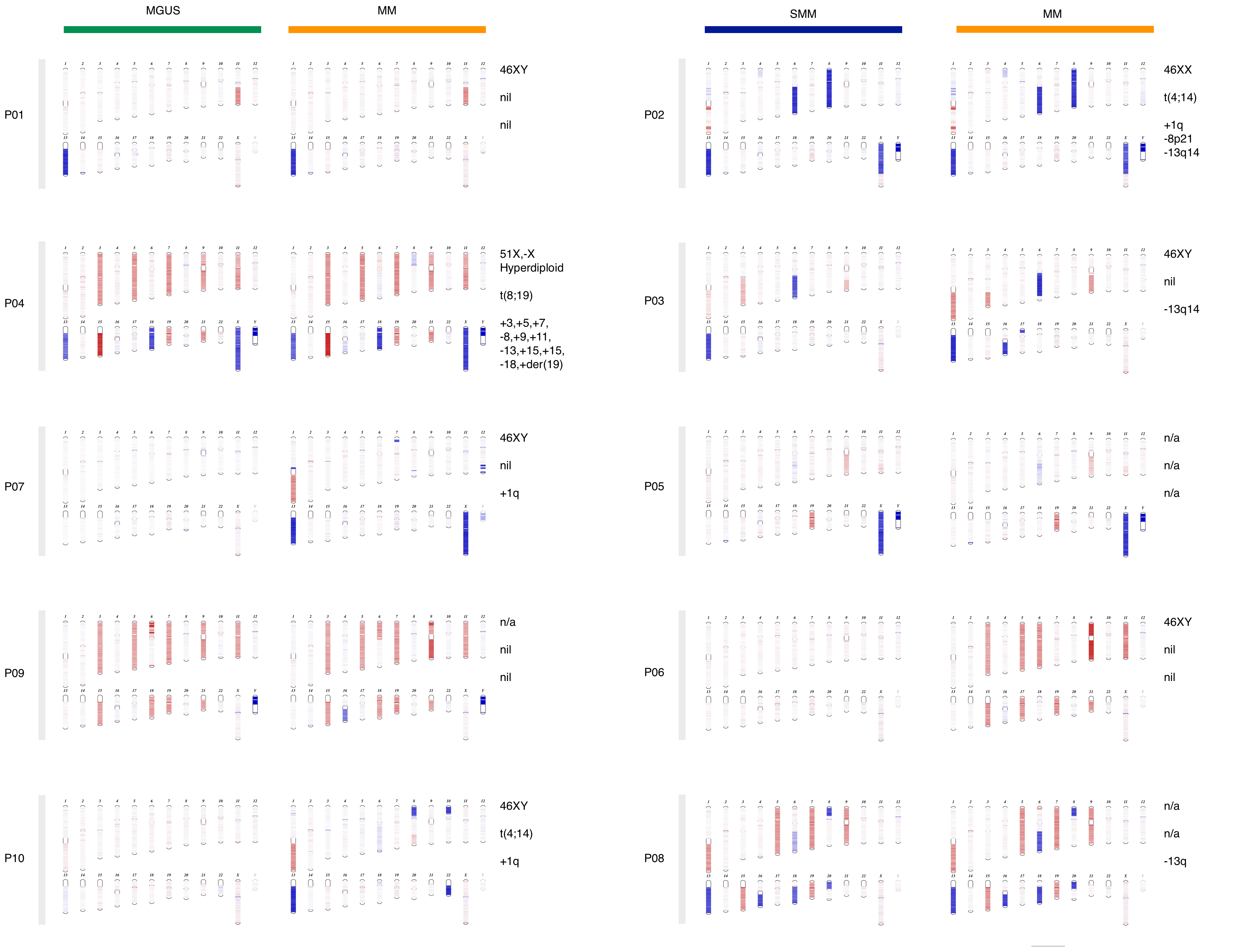


**Supplementary Figure 4. A comparison of cytogenetic abnormalities with virtual karyotypes.** Virtual karyotypes were generated from genome-wide copy number changes inferred from whole exome sequencing data at both MGUS/SMM and MM stages. Chromosomal copy amplifications are illustrated by red, while copy deletions are shown in blue. Several patients exhibit very similar karyotypes at both MGUS/SMM and MM. In some patients, hyperdiploidy is present at MGUS/SMM and is undetected by standard cytogenetics even at MM (molecular cytogenetics results listed to the right of each patient figure). Nil represents parameter not being present. N/A represents that data was not available.
