# Supplementary figures and images for: Subclonal evolution in disease progression from MGUS/SMM to multiple myeloma is characterised by clonal stability
Source: Leukemia. 2018 Jul 25;33(2):457–68. doi: 10.1038/s41375-018-0206-x (PMC6365384; doi:10.1038/s41375-018-0206-x)

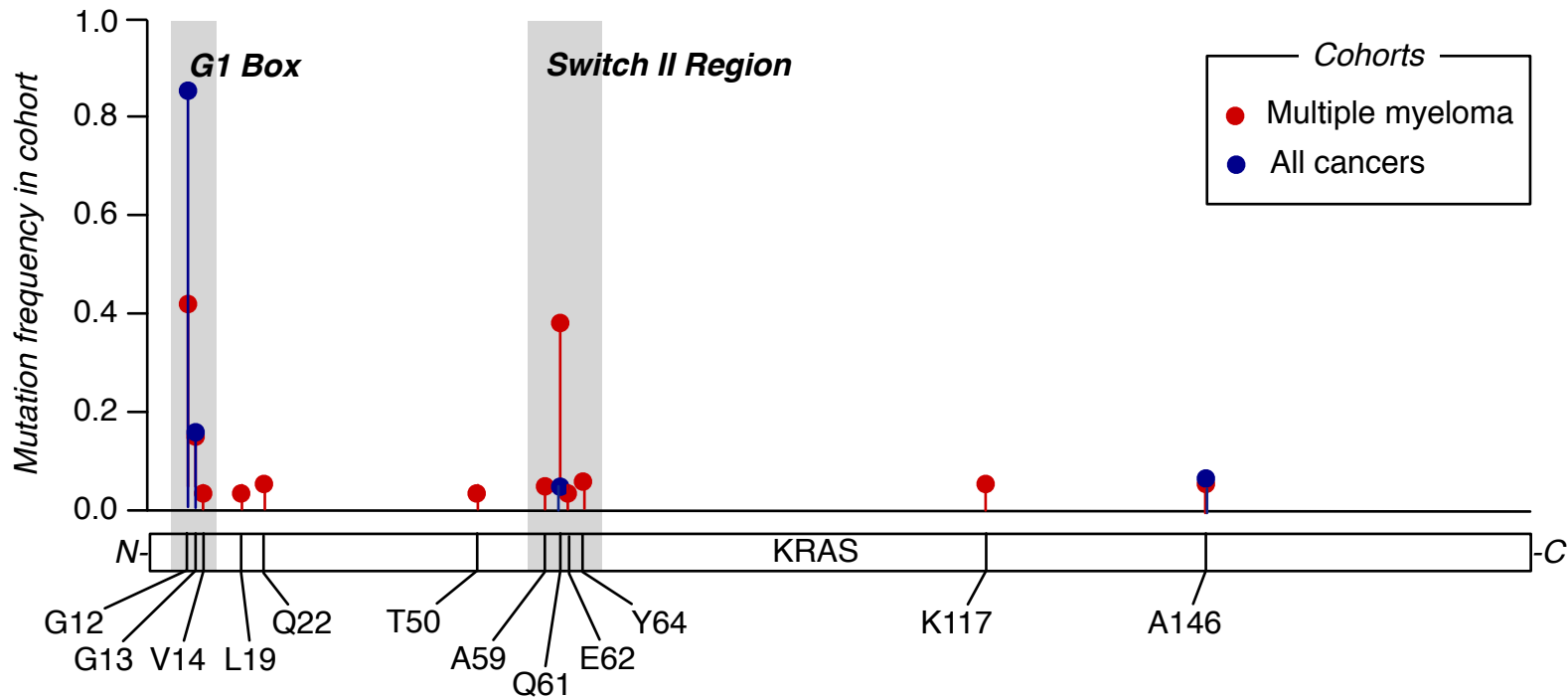

Supplement: Supplementary file 2 — Supplementary Figure 1 [file 41375_2018_206_MOESM2_ESM.pdf]

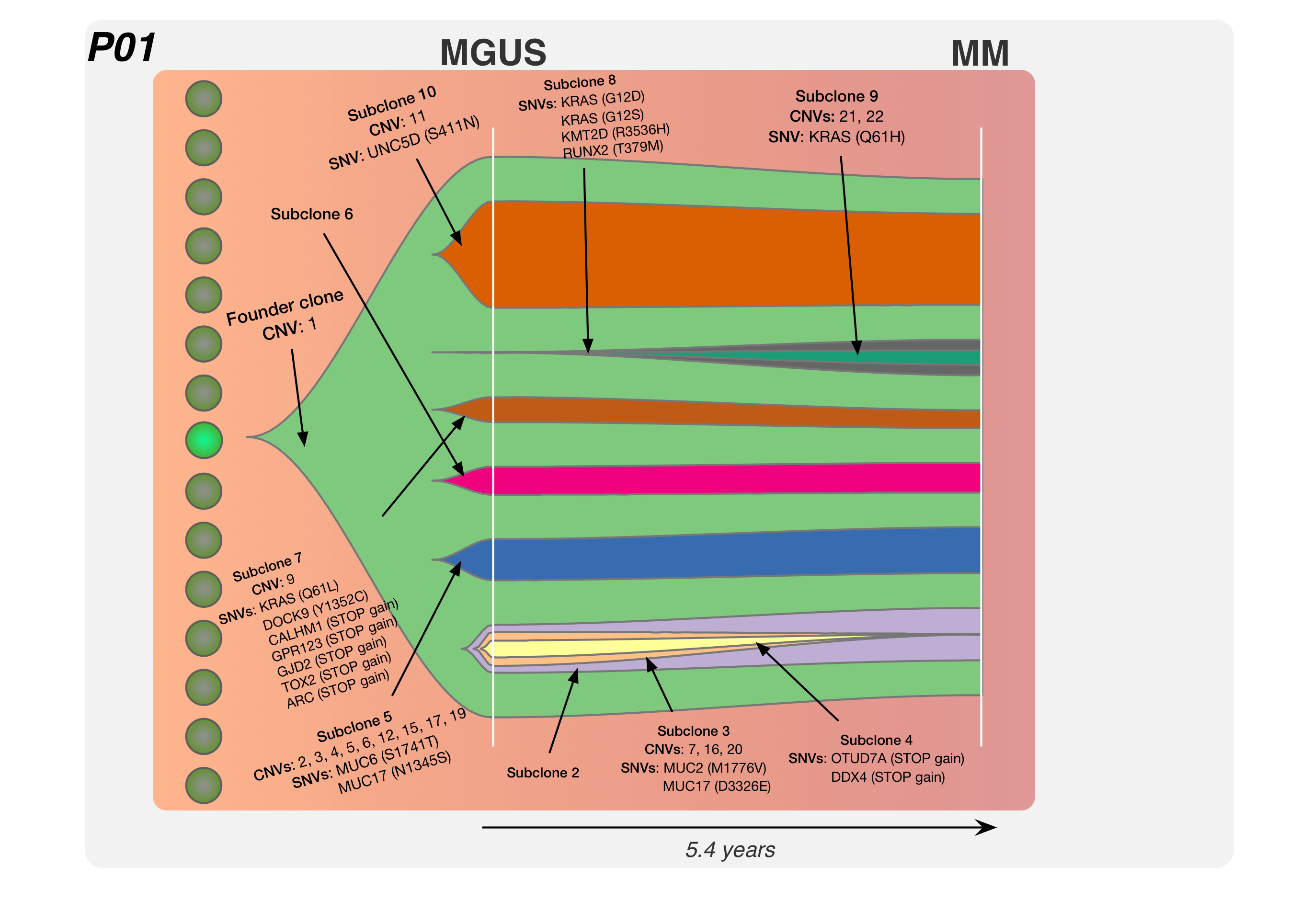

Supplement: Supplementary file 3 — Supplementary Figure 2a [file 41375_2018_206_MOESM3_ESM.jpg]

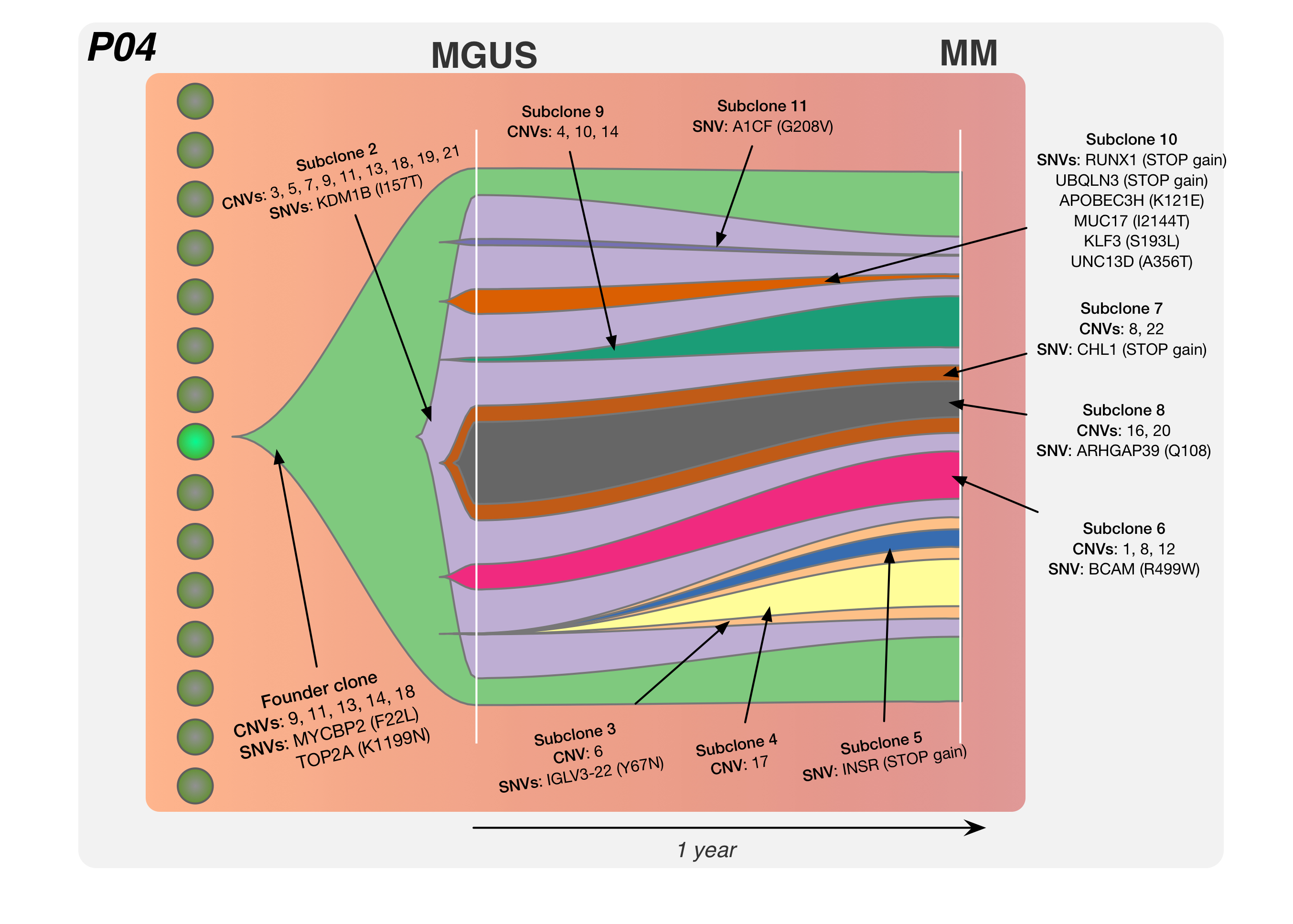

Supplement: Supplementary file 4 — Supplementary Figure 2b [file 41375_2018_206_MOESM4_ESM.jpg]

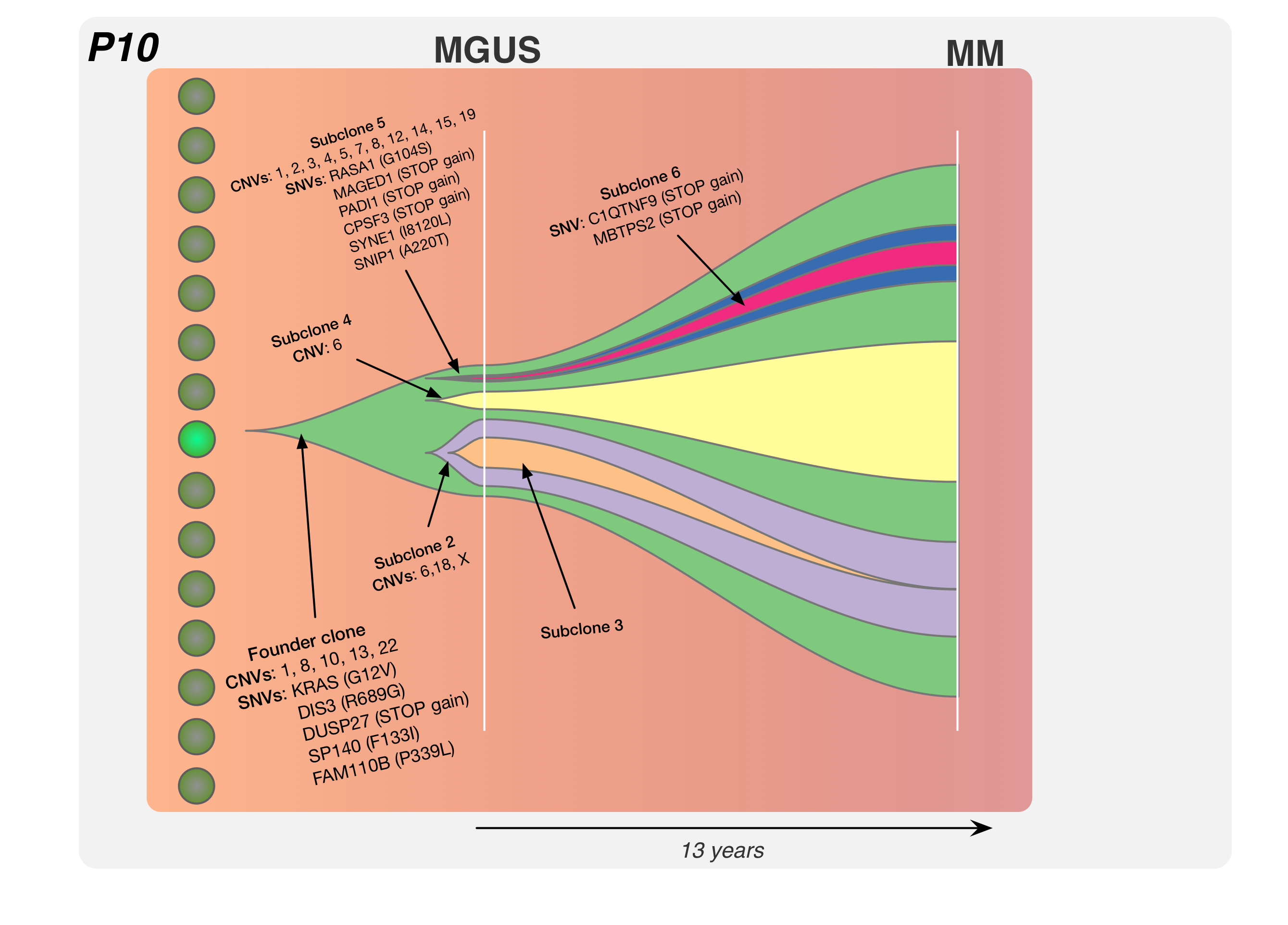

Supplement: Supplementary file 5 — Supplementary Figure 2c [file 41375_2018_206_MOESM5_ESM.jpg]

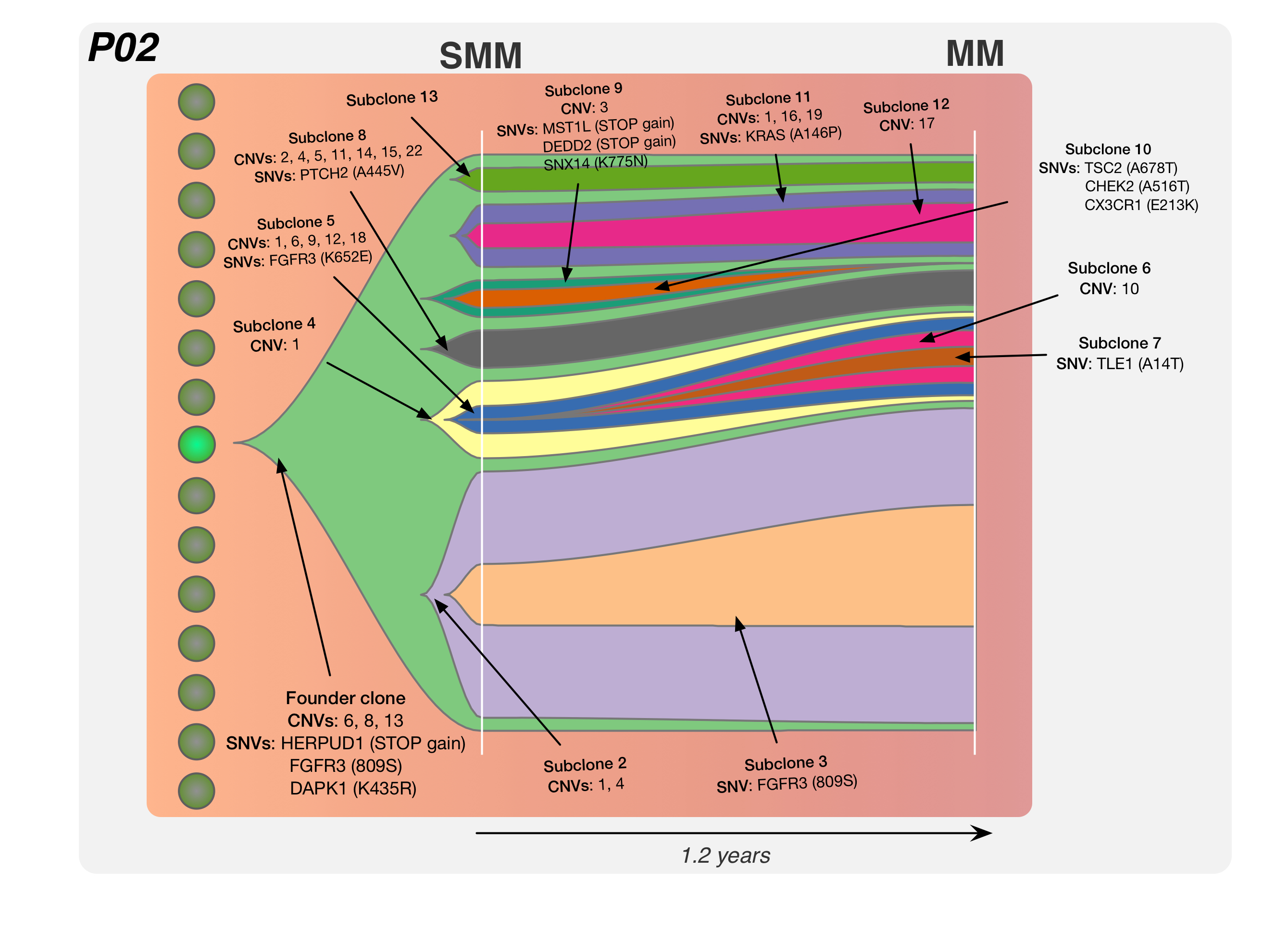

Supplement: Supplementary file 6 — Supplementary Figure 3a [file 41375_2018_206_MOESM6_ESM.jpg]

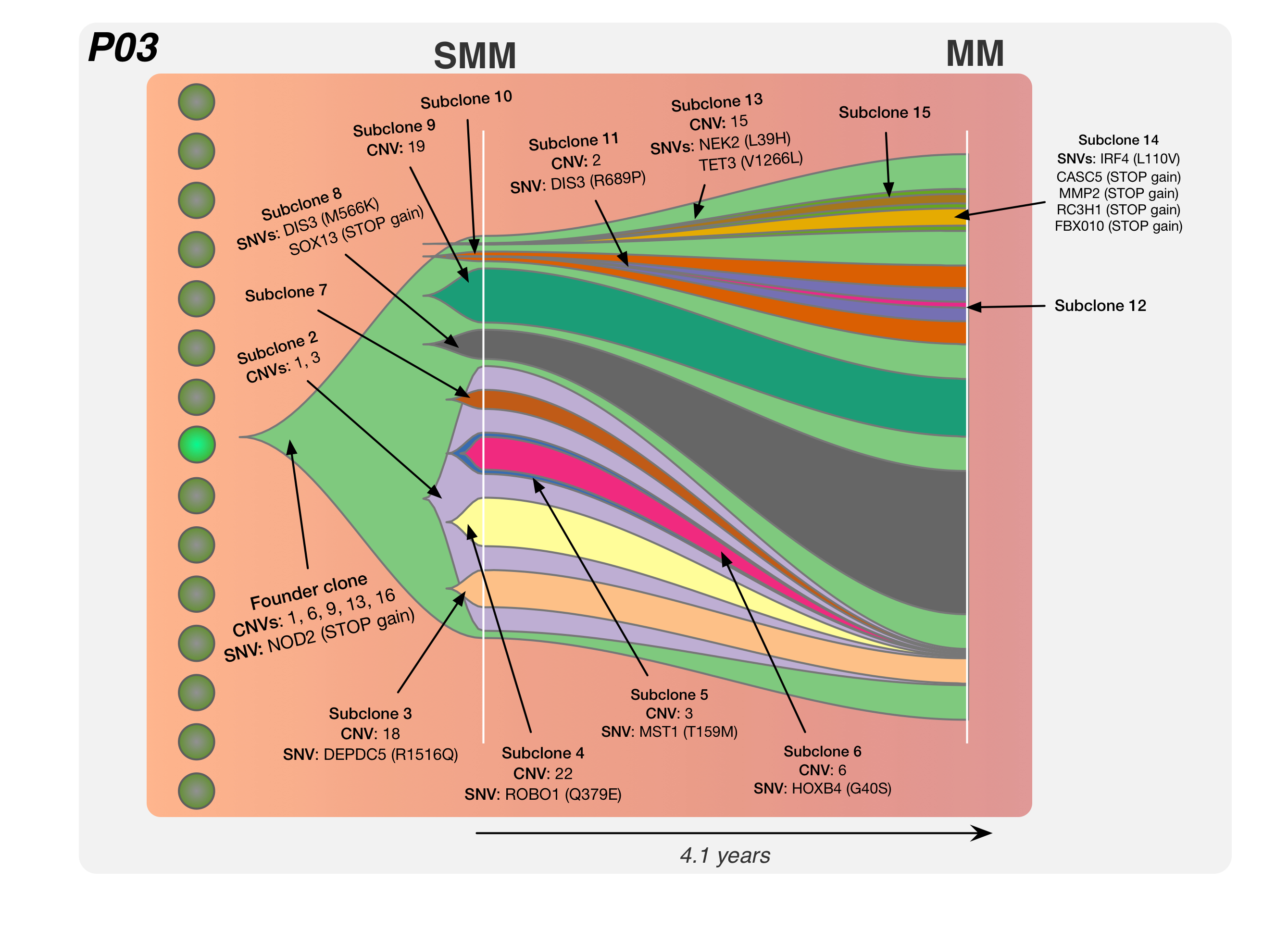

Supplement: Supplementary file 7 — Supplementary Figure 3b [file 41375_2018_206_MOESM7_ESM.jpg]

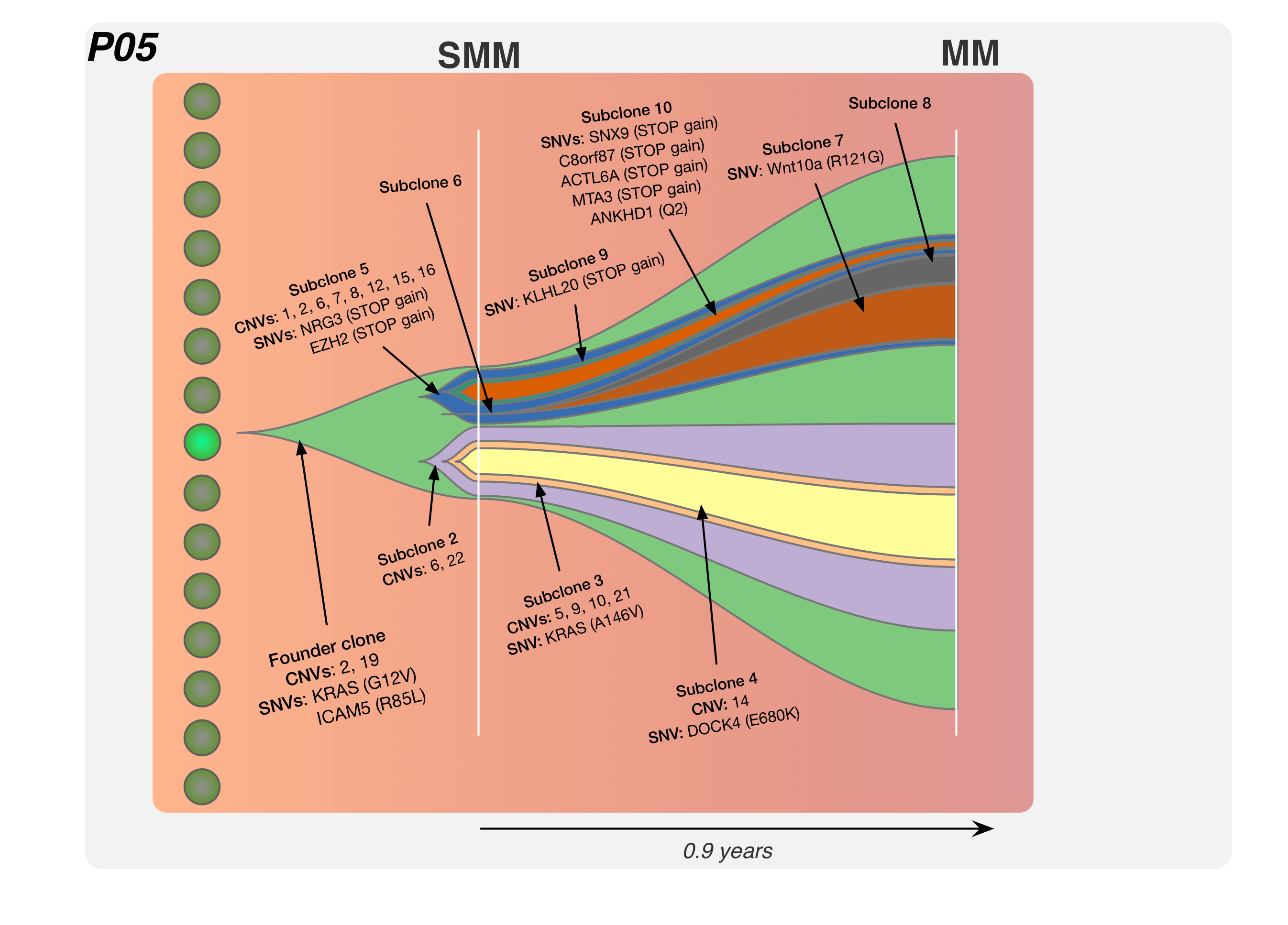

Supplement: Supplementary file 8 — Supplementary Figure 3c [file 41375_2018_206_MOESM8_ESM.jpg]

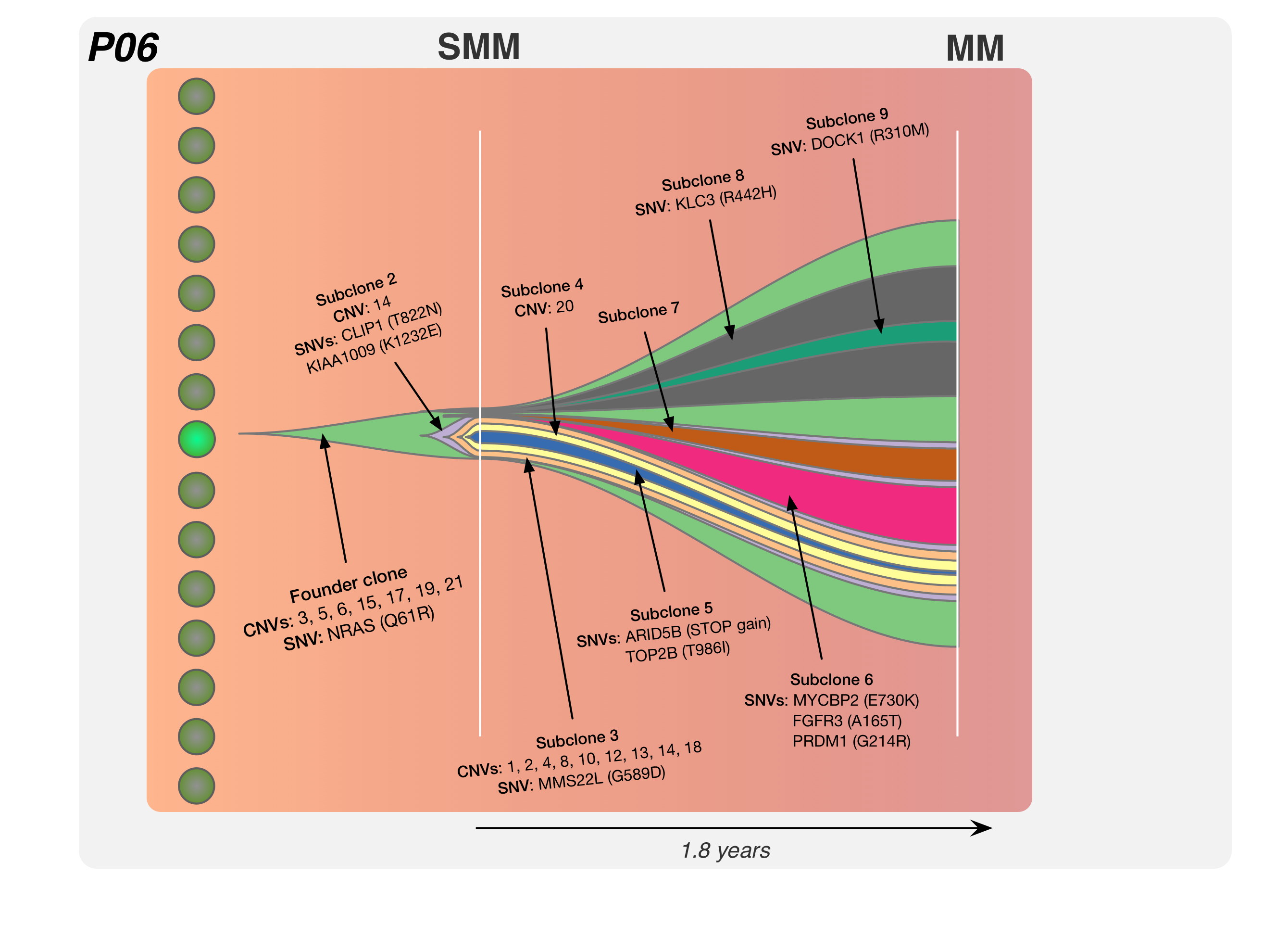

Supplement: Supplementary file 9 — Supplementary Figure 3d [file 41375_2018_206_MOESM9_ESM.jpg]

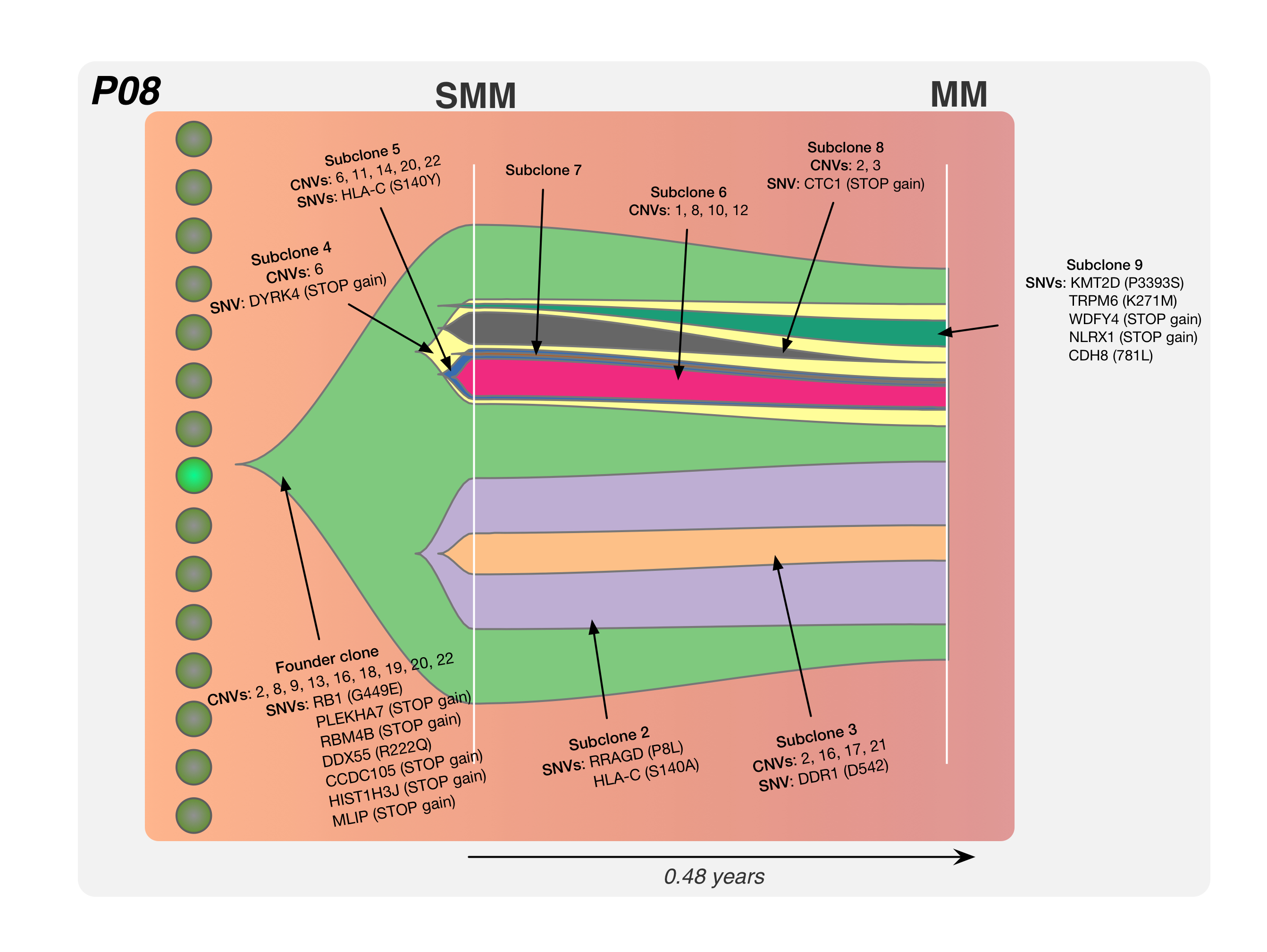

Supplement: Supplementary file 10 — Supplementary Figure 3e [file 41375_2018_206_MOESM10_ESM.jpg]

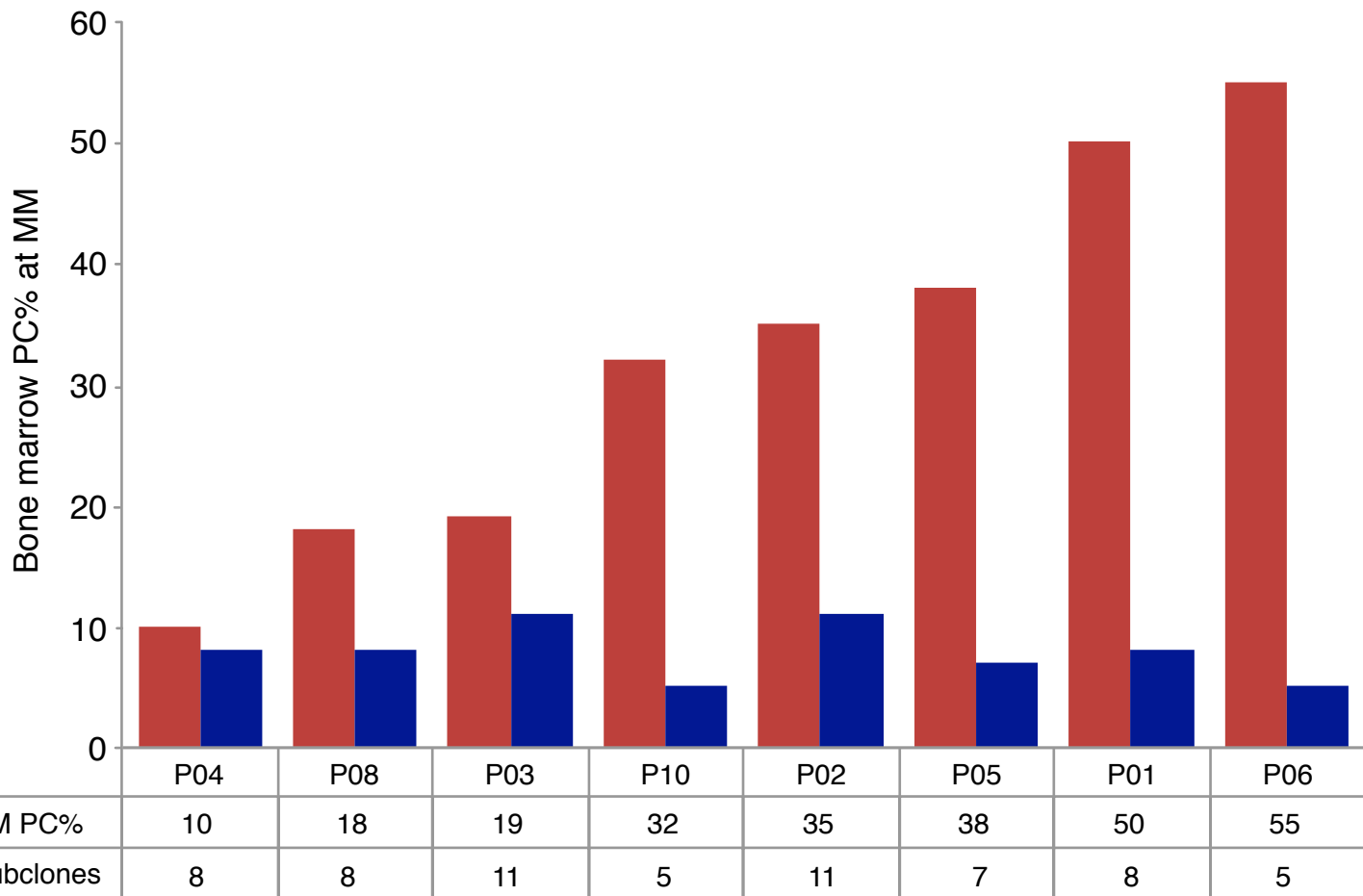

Supplement: Supplementary file 11 — Supplementary Figure 4 [file 41375_2018_206_MOESM11_ESM.pdf]

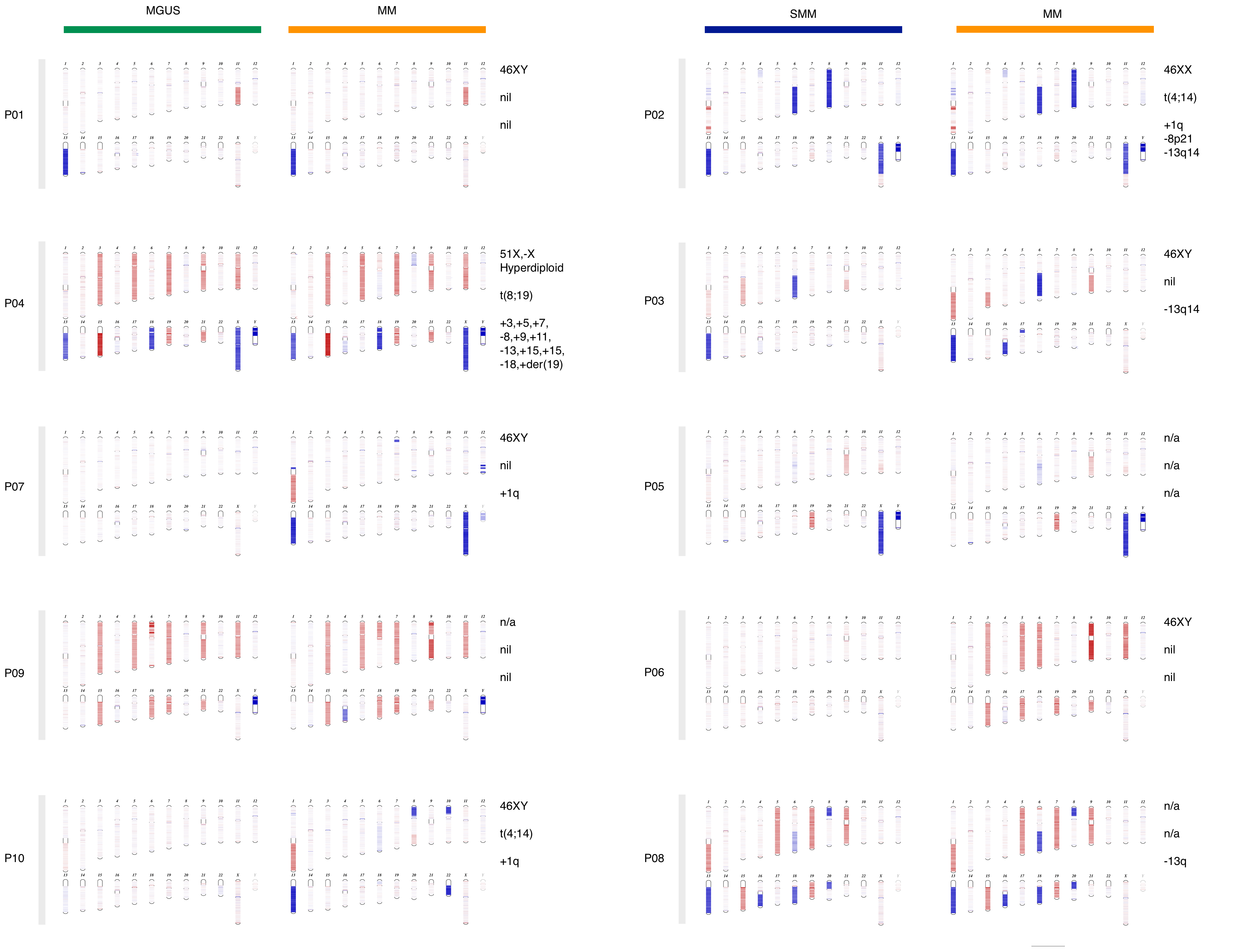

Supplement: Supplementary file 12 — Supplementary Figure 5 [file 41375_2018_206_MOESM12_ESM.png]
